# Cyanobacteria: A Promising Source of Antifungal Metabolites

**DOI:** 10.3390/md21060359

**Published:** 2023-06-14

**Authors:** Samuel Cavalcante do Amaral, Luciana Pereira Xavier, Vítor Vasconcelos, Agenor Valadares Santos

**Affiliations:** 1 Laboratory of Biotechnology of Enzymes and Biotransformation, Biological Sciences Institute, Federal University of Pará, Belém 66075-110, Brazil; lpxavier@ufpa.br; 2 CIIMAR/CIMAR, Interdisciplinary Centre of Marine and Environmental Research, Terminal de Cruzeiros do Porto de Leixões, University of Porto, 4450-208 Matosinhos, Portugal; vmvascon@fc.up.pt; 3 Departamento de Biologia, Faculdade de Ciências, Universidade do Porto, Rua do Campo Alegre, Edifício FC4, 4169-007 Porto, Portugal

**Keywords:** cyanobacteria, secondary metabolites, peptides, alkaloids, antifungal agents, action mechanism

## Abstract

Cyanobacteria are a rich source of secondary metabolites, and they have received a great deal of attention due to their applicability in different industrial sectors. Some of these substances are known for their notorious ability to inhibit fungal growth. Such metabolites are very chemically and biologically diverse. They can belong to different chemical classes, including peptides, fatty acids, alkaloids, polyketides, and macrolides. Moreover, they can also target different cell components. Filamentous cyanobacteria have been the main source of these compounds. This review aims to identify the key features of these antifungal agents, as well as the sources from which they are obtained, their major targets, and the environmental factors involved when they are being produced. For the preparation of this work, a total of 642 documents dating from 1980 to 2022 were consulted, including patents, original research, review articles, and theses.

## 1. Introduction

The fungi kingdom is one of the most diverse on the planet, containing eukaryotic heterotrophs with distinct life cycles, morphologies, and physiologies. Even though they are known for their importance in the ecosystem as decomposers, as well as for their biotechnological potential in pharmaceutical and food industries, many species have been associated with several diseases both in plants and animals, causing a significant number of deaths and major economic losses [1,2]. The incidence of such illnesses has considerably increased worldwide. Several factors have contributed to this scenario, among them, is the excessive use of immune system-modifying drugs and the drastic increase in hematopoietic stem cell transplants [3,4]. Global warming also seems to be one of the elements involved with the rise in diseases caused by fungi since it has contributed to the selection of organisms more resistant to temperature variations, which acts as a barrier against pathogens in animal bodies [5,6]. 

The antifungal agents are only effective in some appropriate contexts since they possess certain limitations, such as elevated toxicity to patients [7]. Moreover, the low availability of drugs for human use, combined with the limited number of cellular targets of these compounds, have facilitated the emergence of multidrug-resistant strains. Currently, it is possible to observe microorganisms that are resistant to all groups of available antifungal agents. Due to these limitations, the discovery of new fungicidal compounds with different targets is crucial [8]. 

As a result, cyanobacteria have attracted considerable attention. These photosynthetic microorganisms are very diverse and widely distributed in nature, including in extreme environments with high temperatures, radiation, and salt concentrations [9,10]. Survival in such environments is guaranteed by the production of a variety of secondary metabolites, which have been extensively investigated due to their ecological relevance and biotechnological value [11,12,13]. Some of these substances exhibit a broad spectrum of action, targeting both eukaryotic and prokaryotic cells, whereas others are quite specific with regard to their targets, only inhibiting the growth of certain organisms [14,15]. 

The metabolites that cyanobacteria produce are known to have peculiar structural characteristics that are associated with increased bioactivity and stability [16,17,18]. Only a small portion of these compounds have been investigated for their antifungal properties. Within this group, there are many promising molecules with inhibitory potential that are superior to commercial antifungals and capable of killing multi-drug-resistant strains. Filamentous cyanobacteria have been the main source of fungicidal agents. The metabolic pathways employed for the production of such substances are relatively complex, involving several steps and different enzymes [19]. 

Although the antifungal activity of cyanobacteria has been explored in some reviews, most of them do not address this issue in depth [20]. Usually, some examples of metabolites are given without many details about their origin and regulation [21]. Most of the works focused on the description of antifungal compounds in these microorganisms are limited to their chemical nature and activity [19]. The mechanism of action of these metabolites is generally overlooked, is little discussed, or when explored, it is restricted to a chemical class [22]. This review is dedicated to describing the main antifungal components obtained from cyanobacteria, as well as their cell targets, and factors involved in their regulation, in order to comprehensively cover this topic. A total of 642 publications were consulted for the preparation of this study, dating from 1980 to 2022. Science Direct and Google Scholar were employed as search engines, utilizing the key words “cyanobacteria” AND “fungicide OR antifungal”. Publications involving the identification and isolation of natural products of cyanobacterial origin were prioritized. Reviews were utilized to complement the data. 

## 2. Methodology

For the preparation of this work, a total of 642 documents from 1980 to 2022 were obtained and preselected from Science Direct and Google Scholar; the key words “cyanobacteria” AND “fungicide OR antifungal” were used as search terms. In some cases, the names of the individual compounds were separately investigated. Publications focused on the discovery of new secondary metabolites, their action mechanisms, and regulation; these publications were prioritized over those that possessed too many limitations for use in an evaluation. Data concerning metabolite structure, target organisms, activity, identification and source of the producing cyanobacterium, regulatory factors, and cellular effect were recorded from these documents. 

In the analysis of the main genus responsible for antifungal compound production, primary metabolites, such as phycobiliproteins, enzymes, and polysaccharides, were not included since the production of such metabolites is a common feature among all cyanobacteria. 

### Antifungal Metabolites from Cyanobacteria

From the last century onward, the fungicidal synthesis of cyanobacteria has been reported and it has been found to contribute to the maintenance of living beings on planet Earth. Many of the interactions of cyanobacteria with other organisms are mediated through these metabolites, which may act as defensive mechanisms against predators and parasites [23]. Fungi are known to be one of the main groups responsible for the population control of cyanobacteria, mainly those belonging to the chytrid group [24]. The ability of this pathogen to infect these photosynthetic microorganisms can be attributed to the presence of chemotactic zoospores and the development of rhizoids that are capable of finding the target and extracting the nutrients, respectively [25]. The molecular repertoire of each cyanobacterial strain can explain the different susceptibility levels in the phylum [26]. Some of these compounds can inhibit vital processes of the fungi, thus preventing the infection. Moreover, they can also target other organisms, such as some bacteria and cyanobacteria, which can act as predators and competitors, respectively [27]. 

**Figure 1:**
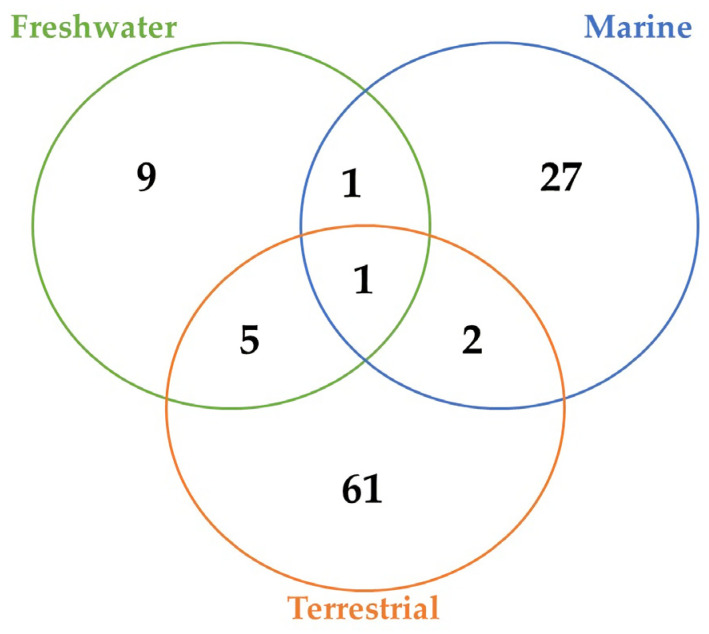
The number of metabolites with antifungal properties identified and shared among strains of cyanobacteria from different environments, including environmental samples.

Over the course of our research project, we found a total of 106 secondary metabolites of cyanobacterial origin with antifungal activity, together with some enzymes, phycobiliproteins, and polysaccharides. Terrestrial strains were the main source of these compounds (Figure 1). The majority of the molecules identified in these cyanobacteria were hapalindole-type alkaloids. These nitrogen-containing compounds never occur alone. They are produced as a mixture of various other alkaloids belonging to the same group. Thus, it is very common to observe the production of various hapalindole-type alkaloids in a single cyanobacterium [28]. The terrestrial ecosystems, compared with others, usually show a higher level of fluctuation, which is representative of a remarkable stress level. The addition of new gene families is a usual strategy employed by cyanobacteria that are isolated from these places, and it contributes to a larger genome in relation to marine and freshwater strains. Certain incorporated genes are associated with the production of natural products [29]. Norharmane was the only product detected in the three types of environments. Cyanobacteria from freshwater and terrestrial environments shared the production of five metabolites, which included members of the Carbamidocyclophane, Scytophycin, and Laxaphycin families. Of the 31 molecules obtained from marine strains, only 2 and 1 are common with terrestrial and freshwater strains, respectively. 

**Figure 2:**
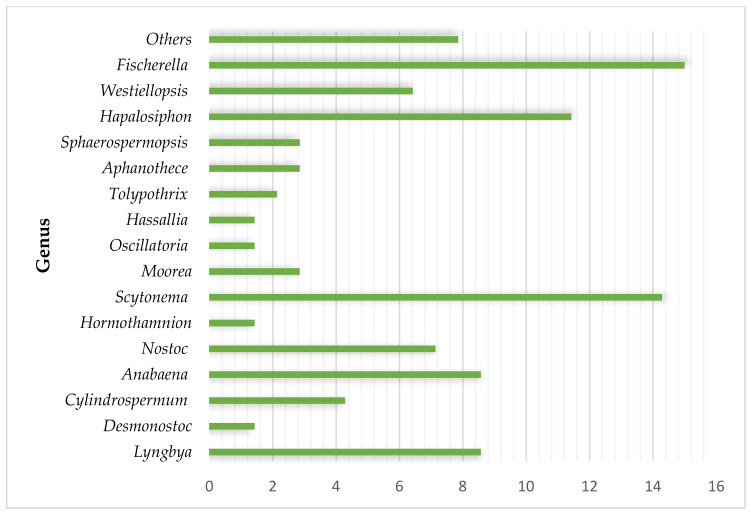
Percentage of compound in each genus.

The metabolites identified here belong to 11 different groups: peptides, phycobiliproteins, enzymes, carbohydrates, fatty acids, alkaloids, polyketides, macrolides, phenolic compounds, terpenoids, and polymers. Peptides and alkaloids were the dominant classes with 40 and 36 representatives, respectively. The comparison between the activity of each metabolite with the control group allowed us to identify various peptides and macrolides that exhibited superior activity to the commercial drugs. Carbohydrates and phycobiliproteins showed reduced bioactivity. Taking only secondary metabolites into account, approximately 27 genera, 17 families, and 6 orders (Synechococcales, Oscillatoriales, Nostocales, Chroococcales, Leptolyngbyales, Geitlerinematales) were documented as producers of antifungal metabolites. The genus *Fischerella* and *Scytonema* produced the highest number of metabolites (Figure 2). 

## 3. Chemical Classes of Metabolites

### 3.1. Peptides 

The majority of antifungal metabolites originating from cyanobacteria are produced in a nucleic-acid-free environment wherein modular multienzyme complexes, known as nonribosomal peptide synthetases (NRPSs) and polyketide synthases (PKS), are present [13,30]. These pathways are capable of producing an enormous variety of structures that differ significantly in terms of their biological activity. Cyanobacterial peptides created from the combination of these pathways, or that are individually produced by NRPSs, normally possess atypical features, such as some chemically modified amino acids produced via methylation [18], cyclization [31], halogenation [17], and dehydration [11,25], as well as the presence of β- and D- amino acids [11]. 

#### 3.1.1. Peptides with β-Amino Acids 

**Figure 3:**
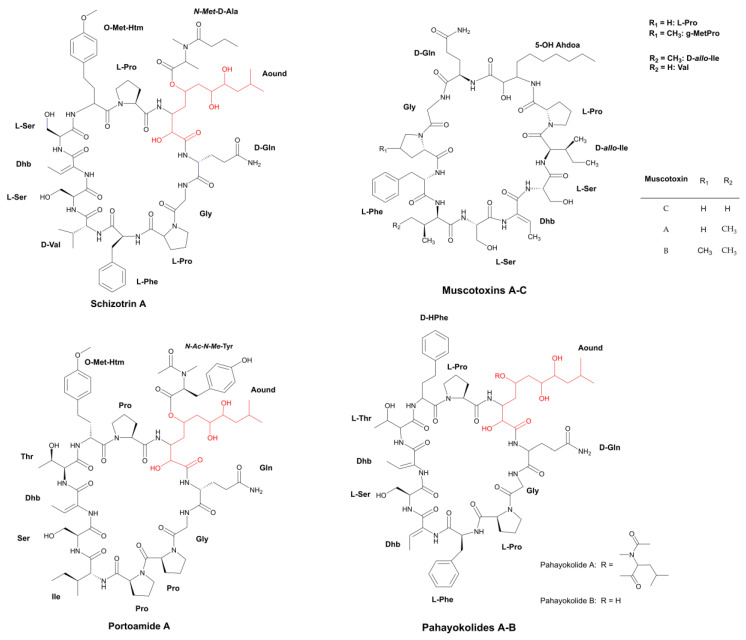
Chemical structure of some antifungal cyanopeptides with β-amino acids.

**Figure 4:**
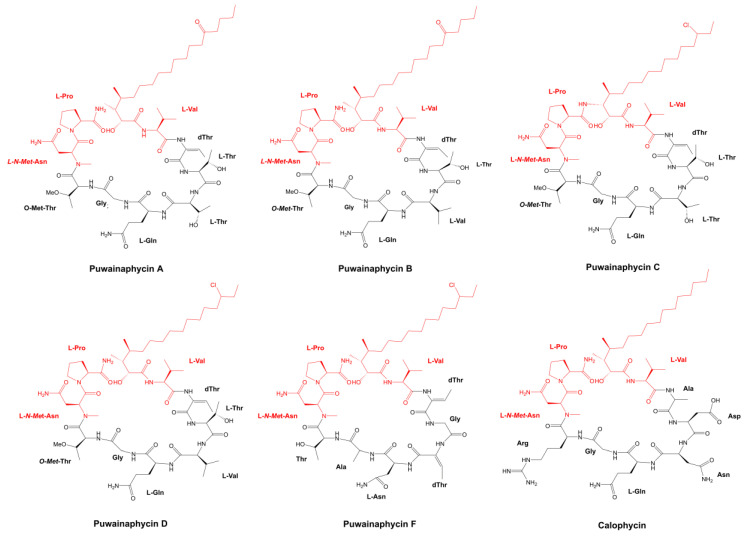
Similarity (red) and differences between the members of the Puwainaphycin family and the cyclopeptide, Calophycin.

The incorporation of β-amino acids into the peptide chain can improve biological activity and enhance resistance to hydrolysis and temperature variations [32]. These modified amino acids have been encountered in some fungicidal cyclopeptides extracted from cyanobacteria, such as the metabolites Schizotrin A (Schiz A) and Pahayokolides A and B (Pahakos A and B) (Figure 3) [33,34]. Both metabolite groups share the β-amino acid 3-amino-2,5,7,8-tetrahydroxy-10-methyl decanoic acid (Aound/Athmu) [33,34]. Other metabolites with this modified amino acid include the algaecides Portoamides (Figure 3), which can be found in the biomass and exudate of the strain, *Oscillatoria* sp. LEGE 05292 [35]. Schiz A is an undecapeptide isolated from the soil cyanobacterium, *Schizothrix* sp. IL-89-2 (Table 1); it possesses the ability to precisely work against the fungi *Saccharomyces cerevisiae*, *Candida albicans*, *Candida tropicalis*, *Rhodotorula mucilaginosa*, *Sclerotium rolfsii*, *Rhizoctonia solani*, *Fusarium oxysporum,* and *Colletotrichum gloeosporioides* (Table 2) [33]. The Proline residue (Pro) segment connected to the Athmu motive in this oligopeptide seems to be required for biological activity to occur [33]. Proline residue attached to a β-amino acid is also a common feature of the oligopeptides, Puwainaphycin C (Puwa C) and Calophycin (Figure 4) [36,37], the latter of which is a decapeptide obtained from the freshwater strain *Calothrix fusca* EU-10-1. Its action spectrum is quite broad, exerting an inhibitory effect against a variety of fungi (Table 2). Its Pro residue is linked to 3-amino-2-hydroxy-4-methylpalmitic acid (Hamp) [36].

Pahakos (Figure 3) are larger cyclopeptides obtained from the freshwater cyanobacterium, *Lyngbya* sp. 15-2 (Table 1) [38]. To date, only four Pahakos (A–D) have been identified. Pahako B has been identified as one of the minor constituents of the extract, and it has the same polyketide skeleton as Pahako A, differing only in terms of its *N-acetyl-N-methyl* leucine unit, which is removed from its structure through the cleavage of an ester bond. Pahakos C and D are conformers of Pahako A, which is similar to Portoamide A; they also exhibit anti-algal activity and cytotoxicity in a varied number of cancer cell lines. Regarding its antifungal potential, only *S. cerevisiae* was tested as an indicator. A paper disk loaded with 30 µg of Pahako A provided a 20 mm zone of inhibition for this yeast [38]. 

Muscotoxins A–C (Muscos) (Figure 3) are additional lipopeptides with elevated similarities to Schiz A. They are formed when a variant of a β-amino acid (3-amino-2,5-dihydroxydecanoic acid) links with an aliphatic chain composed of ten residues, of which, seven are identical to those found in Schizotrin A, whereas the other three possess an equivalent polarity (Figure 3) [39]. Although the differences between the Muscos normally occur in only one amino acid, they significantly impact the minimal dosage required for the manifestation of antifungal activity against the phytopathogen, *Sclerotinia sclerotiorum*. For instance, the substitution of isoleucine (Ile) with valine (Val) at the sixth position in Muscotoxin C. Furthermore, the remaining Muscos results in the total abolishment of antifungal activity, and the methylation of a proline residue in Muscotoxin B is responsible for the increase in activity. Due to the low yield of Muscos B–C, only Muscotoxin A has a determined spectrum of activity, demonstrating moderate and strong antifungal activity against a variety of fungi (MIC ranged from 0.58 to 75 µg mL^−1^) and restricted bioactivity in bacteria [40].

Initially isolated from the Hawaiian terrestrial cyanobacterium, *Anabaena* sp. BQ-16-1, Puwainaphycins (Puwas) (Figure 4) are a cyclic decapeptides class formed when a β-amino fatty acid is connected to a nine-membered peptide ring. The elements belonging to this family can be clearly distinguished by variances in the length and functional unit occurrences (such as ketone and chlorine) in the fatty acid chain [37]. Their names are derived from the site of the Punchbowl National Cemetery, which is mainly known as Puowaina in Hawaiian [112]. Since the discovery of Puwainaphycins A–E by Moore and his research group, other analogous compounds have been described in freshwater cyanobacteria, such as those *Aphanizomenon gracile* strains (PMC638, 644, and 649) investigated by Halary and colleagues, and the Brazilian *Aliinostoc* strains (535 and 548) isolated from the alkaline lake, Salina Verde [113,114].

The soil cyanobacterium, *C. alatosporum* CCALA 988, is currently the model organism for the study of Puwas. In addition to Puwas F (Figure 4) and G (the only difference between them is situated in the fourth position), the microorganism harbors nearly 25 Puwa F and G congeners [41,115,116]. The production of some of these congeners is shared by other *Cylindrospermum* strains (CCALA 993 and 994) and *Anabaena* sp. UHCC-0399 [115]. The synthesis of various Puwas with a single strain, containing only one operon for oligomers, can be justified with the promiscuity of the fatty acyl-AMP ligase (FAAL) starter unit and the multi-specificity of several domains [41]. Puwa F bioactivity has been tested against a panel of microorganisms, which produced no effects against either gram-negative or gram-positive bacterial strains. Moreover, it exerted antagonistic activity for the yeasts, *C. albicans* HAMBI 261 and *S. cerevisiae* HAMBI 1164, with a MIC (Minimum inhibitory concentration) of 5.5 µM (6.3 µg mL^−1^) (Table 2) [79]. 

**Figure 5:**
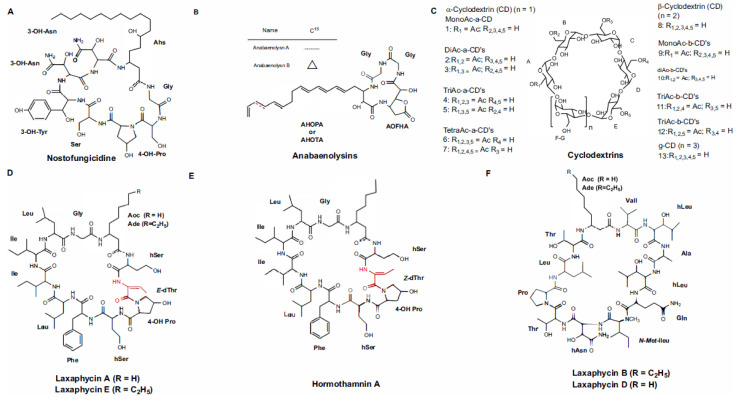
Fungicide isolated from cyanobacteria. (**A**) Nostofungicidine. (**B**) Anabaenolysins A and B. (**C**) Cyclodextrin variants at different acetylation levels. (**D**) Laxaphycins A and E. (**E**) Hormothamnin A. (**F**) Laxaphycins B and D.

Nostofungicidine (Figure 5) is another antifungal cyclic peptide of cyanobacterial origin with a β-amino acid. This lipopeptide is obtained from the methanolic extract of the field-grown terrestrial cyanobacterium, *N. commune* (Table 1), and it exhibited bioactivity against *A. candidus* (Table 2). In addition to the β-amino acid 3-amino-6-hydroxy stearic acid (AHS), its structure harbors various hydroxy-derivatives (Figure 5) [45].

Anabaenolysins (Abls) are structurally comparable to Nostofungicidine, displaying a long tail formed by a β-amino fatty acid connected to a peptidic macrocycle composed of two proteinogenic amino acids and the unusual amino acid 2-(3-amino-5-oxytetrahydrofuran-2-yl)-2-hydroxyacetic acid (AOFHA) (Figure 5) [46]. An initial screening for cytolytic substances, involving nearly 30 Baltic Sea benthic cyanobacteria, led to the acquisition of Abls A–B from two strains of *Anabaena* (XPORK 15F and XSPORK 27C) [46]. A structural comparison of both demonstrates that those molecules are distinguishable only because of an unsaturated β-amino acid at C-15, which confers the presence of a conjugated dienic structure to Ana A, which is not found in Anabaenolysin B. The strain XPORK 15F harbors eight additional Abls; the main differences between them are the length and number of double bonds present in the hydroxyamino fatty acid. Due to the abundance of these lipopeptides in the *Anabaena* biomass, it is believed that they are of great importance for the survival of producing cyanobacteria [46]. 

Abls produce little antifungal activity against *C. albicans*, whereas the crude extract containing these lipopeptides has been shown to have improved efficacy in this respect. This contrast is due to the presence of cyclodextrins (Figure 5) in the extract, which are co-produced with Abls and act in synergy with this lipopeptide group [47]. The mechanism by which cyclodextrins act, in terms of its ability to enhance Abls’ productivity, has not been fully elucidated. However, the presence of various types of cyclodextrins with distinct properties in the extract suggests the existence of multiple pathways [47]. The co-occurrence of a hydrophilic external surface formed by the hydroxyl groups and an internal hydrophobic portion makes the cyclodextrins excellent solubilizers with enormous potential as carriers in the pharmaceutical and food industries [117]. Alpha-dextrin is the most recurrent dextrin of the *Anabaena* that are isolated from the Baltic Sea; it is likely to be involved in increasing the solubility of Abls through the formation of an inclusion complex with a hydrocarbon chain. Collectively, the small amount of β-dextrin, a by-product from Abls-producing *Anabaena* strains, is most likely to be involved in the fungal ergosterol capture from the plasmatic membrane, thus resulting in the loss of membrane integrity [47].

Synergistic interactions have also been documented in cyclopeptides belonging to the Laxaphycins (Laxas) family, which were originally extracted from the non-polar extract of the terrestrial cyanobacterium *A. laxa* UH FK-1-2 (Table 1) [48,118]. The joint antifungal effects of several Laxas are more pronounced than the individual action of each one. In certain situations, the biological activity of the HPLC fraction containing the separated peptide is absent [48,118]. Interestingly, synergism occurs mainly in fractions containing Laxas (Table 2) with distinct molecular weights, such as Laxas A and B, which are the main peptides present in the extract. The antiproliferative property of Laxas was initially demonstrated for the fungi *A. oryzae*, *C. albicans*, *P. notatum*, *S. cerevisiae*, and *T. mentagrophytes*, as well as the tumor cell lines, KB (epidermoid carcinoma) and LoVo (colorectal adenocarcinoma) [118]. Among these microorganisms, *A. oryzae* was the most sensitive to the action of these cyanopeptides. The purified Laxa B exhibits a MIC value of 45.8 µM mL^−1^ for this fungus; however, when combined with 8.1 µM of Laxa A, only 4.6 µM is needed to produce the same effect [118]. A similar phenomenon was also reported by Bonnard and co-workers in 1997 for *C. albicans* using Laxa A and B [50]. In contrast with the above results, Pennings and colleagues demonstrated that the purified Laxa A was bioactive, and capable of deterring the parrotfish, *Scarus schlegeli*, the sea urchin, *Diadema savignyi*, and the crabs, *Leptodius* sp. from feeding [119]. These data are consistent with those of Dussault and colleagues, who demonstrated the bioactivity of Laxa A using various Gram-positive bacteria [120].

The structural investigation of Laxas allowed us to split the members of this family into two classes based on the amino acid distribution and number. The former group includes all those Laxas that are structurally related to Laxa A (Figure 5), a cyclic undecapeptide whose sequences reveal an interesting segregation between hydrophobic and hydrophilic residues [48]. The second group is formed by Laxas originating from the dodecapeptide Laxa B, which, in contrast to Laxa A, contains alternating hydrophobic and hydrophilic amino acids. Currently, the Laxas family comprises nearly 42 lipopeptides [49,52,53,121,122,123,124,125]. All are characterized by a diversity of exotic amino acids, such as β-amino acids with a short linear chain of 8 or 10 carbons, and 4-hydroxyproline, hydroxythreonine, and hydroxyasparagine [53].

Laxas have been documented in cyanobacteria of diverse origin, which indicates its horizontal transfer among members of the phylum or the presence of a common ancestor. Among the species reported as producers are *L. majuscula* [50], *A. torulosa* [117], and *H. enteromorphoides* [119]. Its biotransformation was primarily reported by Alvariño and co-workers (2020) in the herbivorous gastropod *Stylocheilus striatus*, and it is involved in amino acid deletion and ring-opening [124]. Recently, the Laxas biosynthetic pathway was elucidated using the genome of the cyanobacterium, *S. hofmannii* PCC 7110 [52]. *S. hofmannii* PCC 7110 Laxas are called Scytocyclamides, whereas those extracted from *Nostoc* sp. UHCC 0702 receive the name of Heinamides [52,53]. Both have been tested using a panel of bacteria and fungi (Table 2), and they demonstrated a very precise antagonistic activity against the fungus *A. flavus* FBCC 2467 (Table 2) [52,53].

At the same time that Laxas were discovered, Gerwick and his research group elucidated the total structure of the cyclopeptide, Hormothamnin A (Hormo A) (Figure 5), and they isolated it from the marine cyanobacterium, *H. enteromorphoides* [54]. The peptide has a similar structure to Laxa A, differing only in the stereochemistry of the dehydrohomoalanine residue. Interestingly, Hormo A also synergistically works with Laxa B to provide powerful antimicrobial activity [126]. Regarding the antifungal property of Hormo A, the metabolite failed to inhibit *C. albicans* and *T. mentagrophytes*, whereas the HPLC fraction containing Hormos C, D, G, J, or K individuals displayed moderate biological activity against *C. albicans* [77]. Contrary to Laxa A, Hormo A is highly cytotoxic in a diversity of solid cancer cell lines [77]. 

**Figure 6:**
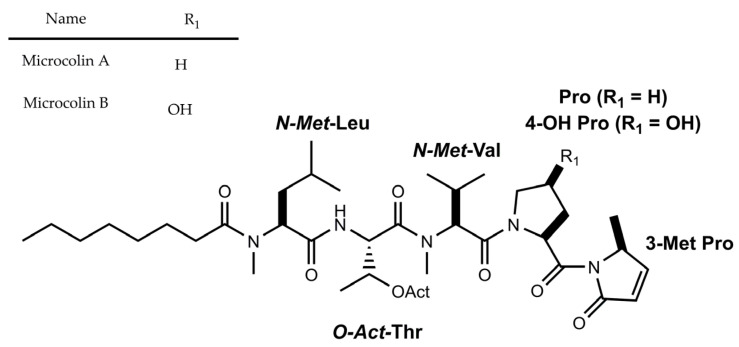
Structure of the peptides, Microcolins A and B.

Thus far, Microcolins (Figure 6) are one of the few antifungal lipopeptides with an acyclic structure that are documented in cyanobacteria [56]. The discovery of Microcolins A–B occurred during an investigation of the immunosuppressive activity of an ethanolic extract in a Venezuelan sample containing the cyanobacterium, *L. majuscula* [56]. Both manifest a very modest antifungal activity in relation to the known antifungal compound, amphotericin B. Their median lethal dose values (LD_50_) for the two strains of marine fungus *Dendryphiella salina* are greater than 200 µg mL^−1^ (250 µM) [56]. In contrast, amphotericin B is capable of reducing the growth of this strain by 100%, at a concentration of 3.13 µg mL^−1^ (3.4 µM) (Table 2) [55].

#### 3.1.2. Peptides with Thiazole and Oxazole Rings

**Figure 7:**
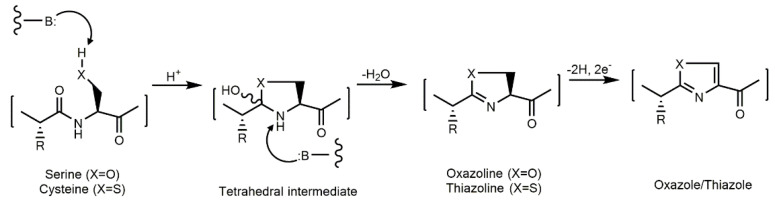
Biosynthesis of oxazole and thiazole rings from the amino acids, Serine and Cysteine, respectively.

Thiazole and oxazole rings are part of certain antifungal peptides identified in cyanobacteria. Metabolites bearing these moieties have been extensively investigated due to their significant pharmacological relevance [127]. Oxazole displays a ring structure with five members, where the first position is occupied by an oxygen atom and the third position is occupied by a nitrogen atom (Figure 7). It can be biologically obtained from the cyclization and oxidation of the following amino acids: threonine and serine (Figure 7) [128]. Thiazole is an oxazole analog that originates from the cysteine amino acid (Figure 7). The oxygen atom in oxazole is replaced by a sulfur atom in thiazole. Both are found mainly in marine organisms [128]. The incorporation of these elements not only modifies the peptide backbone connectivity, but also the interaction with the target molecules [129].

**Figure 8:**
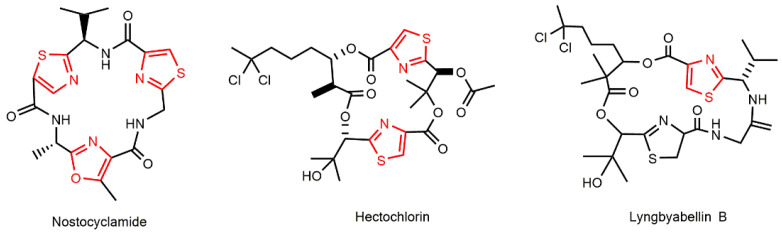
Cyanopeptides with fungicidal properties bearing thiazole and oxazole rings.

Examples of cyclic hexapeptides containing two thiazole residues and one oxazole residue, that are linked by three peptide units, are Nostocyclamide and Nostocyclamide M (Figure 8). These were isolated by Todorova and his research group, and they were classified as allelochemicals from the freshwater cyanobacterium, *Nostoc* sp. 31 (Table 1) [61,62]. A chemical comparison of these metabolites demonstrates that both are almost structurally identical, diverging only by one amino acid residue. In addition to its significant algaecide effect, especially for cyanobacteria, Nostocyclamide has demonstrated very restricted antifungal activity against *S. cerevisiae* (Table 2), some rotifers, and crustaceans [62,130]. Of note, this cyanopeptide has been also found in the cyanobacterium, *A. halophila*, which has a very distinct lifestyle compared with *Nostoc* sp. 31 [131]. 

The chlorinated lipopeptide, Hectochlorin (Figure 8), obtained from the Jamaican *L. majuscula*, also occupies the motif thiazole [132]. This metabolite has been extensively studied due to its moderate activity against a panel of cell lines originating from various tissues, mainly colon, melanoma, and ovarian; it showed an average IC_50_ value of 5.1 µM. Its remarkable antimicrobial potential has been confirmed for the yeast, *C. albicans* ATCC 14053, and several fungi related to crop diseases [76,132]. At a dose of 100 and 10 µg/disk, the lipopeptide provokes a 16- and 11-mm zone of inhibition, respectively, against *C. albicans* (Table 2). Bacteria treated with Hectochlorin were unaffected in terms of [76]. 

The members of the Lyngbyabellins (Figure 8) family are structurally related to Hectochlorin and the natural product, dolabellin, which was first reported in the sea hare *Dolabella auricularia*, a generalist herbivore. With exception of Lyngbyabellin B, all other 17 elements belonging to this group share two thiazole units and one chlorinated β-hydroxy acid [133]. Their antifungal property has hardly been explored; only Lyngbyabellin B has been tested, exhibiting an inhibition zone of 10.5 mm to *C. albicans* ATCC 14053 at 100 µg (Table 2) [76]. Neither the gram-positive nor gram-negative bacteria that were used as targets had their growth blocked in the compound’s presence [76]. In addition to the *Lyngbya* genus, only cyanobacteria belonging to the *Moorea*, *Okeania,* and *Perforafilum* genera. All Oscillatoriales have been documented as sources of these peptides [134,135,136,137]. Considering the relationship between biological activity and structure, the cyclization, the number of chlorine atoms, and the side chain seem to favor the cytotoxicity of Lyngbyabellins [133,135,136]. However, this is not a general rule as some acyclic Lyngbyabellins may still exhibit pronounceable anti-tumor activity; this is likely due to their ability to conform, similarly to the cyclic forms when they interact with the target molecule. Furthermore, in contrast to the results documented for cytotoxicity, the number of side chains appears to slightly diminish the anti-fouling effects of Lyngbyabellins. The evaluation of the potential effects of these molecules, including their antifungal properties, as well as the discovery of new representatives, can better clarify such a relationship [134,136,138].

#### 3.1.3. Lipoglycopeptides

**Figure 9:**
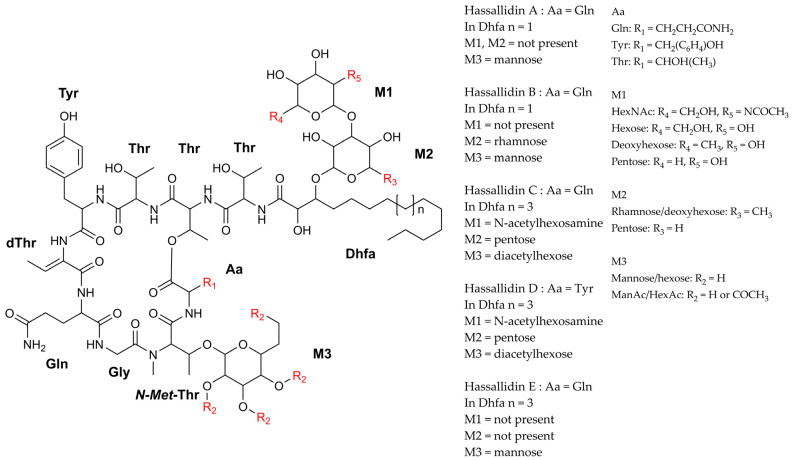
The structure of Hassallidins A–E. Abbreviations: Dhfa (dihydroxy fatty acid), dihydroxy tetradecanoic acid in hassallidin A and B, and dihydroxy hexadecanoic acid in hassallidin C, D, and E.

In 2005, Neuhoff and coworkers reported a new structural type of antifungal agent that was extracted from the epilithic cyanobacterium, *Hassallia* sp. B02-07 (Table 2). The compound received the name Hassallidin A (Has A) (Figure 9), and it consists of an esterified eight-residue cyclic peptide linked to an amino acid, an α, β-dihydroxytetradecanoic acid, and a mannose. Spectrum analyses revealed the presence of the following nonproteinogenic amino acids within the peptide moiety: dehydroaminobutyric acid, D-glutamine, D-Tyrosine, D-Threonine, and D-allo-threonine [68]. One year later, Has B (Figure 9) was discovered in the same strain. It differs from the first family member only in that there is rhamnose in the fatty acid. This modification was responsible for conferring better water solubility to the molecule, but little altered in terms of its anti-fungal properties, which were tested against a panel composed of 16 Candida strains [67]. Subsequently, the genome sequencing analysis of *Anabaena* sp. SYKE748A enabled the discovery of various other variants, not only from this cyanobacterium, which produces at least 40 distinct analogs, but also from other 20 *Anabaena* strains; these were selected from a screening process involving 99 species of the genus. Has C and D were the most abundant compounds. Both shared the same amino acid backbone as Has A and B, differing only in position 10, which was occupied either by glutamine (Has C) or tyrosine (Has D). In this same study, Has biosynthesis was observed in other heterocystous cyanobacteria, such as *C. raciborskii* ATCC 9502 and CS-505, *A. gracile* Heaney/Camb 1986 140 1/1, *Nostoc* sp. 159 and 113.5, and *Tolypothrix* sp. PCC 9009. Contrary to the Has identified among *Anabaena* strains, which exhibit significant structural variation in terms of sugar composition and fatty acid chain length, the Has detected in these strains barely diverged among them. A similar analysis was performed by Shishido and coworkers, who documented the occurrence of 14, 9, 10, and 9 Has variants in *Nostoc* sp. CENA 219, *Anabaena* sp. BIR JV1, *Nostoc calcicula* 6 sf Calc, and *Anabaena* sp. HAN7/1, respectively [69]. Has E was one of the newest members discovered in the benthic cyanobacterium, *P. serta* PCC 8927 (Table 1) [70]. Regarding the antifungal properties of these new congeners, Has D demonstrated a MIC value of 2.8 µg mL^−1^ for *C. albicans* and *C. krusei*, and its linear form gave a value of 36 µg mL^−1^ (Table 2); therefore, this demonstrates the importance of the ring structure in terms of the inhibitory potential of these molecules. A superior MIC value was obtained for Has E (MIC: 32 µg mL^−1^) which was tested using *C. albicans* CBS 562, *C. neoformans* H99, *C. parapsilosis* ATCC 22019, and *C. krusei* ATCC 6258 [69,70]. The absence of an acetylated sugar, or the presence of a very long fatty acid chain, could be a reason for the reduced activity [69,70]. 

Hassallidin gene clusters have been documented in the total genome of several cyanobacteria [70,139,140,141,142]. Some of these clusters are located in the plasmids, as are those encountered in the cyanobacteria, *Aulosira laxa* NIES-50 and *T. tenuis* PCC 7101 [143]. However, the presence of such gene clusters does not guarantee the biosynthesis of these metabolites since some mutations can occur within genes, thus preventing their activity [144]. For example, *Anabaena* sp. 90 harbors the Has gene cluster, but is not capable of producing the metabolite due to a 526 bp deletion in the has *V* gene that occurred between 2003 and 2006. Curiously, through the inactivation of the anabaenopeptilide gene cluster, a genetically modified *Anabaena* sp. 90 maintained the genes necessary for the production of intact Has, though it demonstrated inferior growth when compared with the wild strain. The event indicates that a lower metabolic burden favors cell growth [144]. The cryptic genes possess an important evolutive role due to their potential to increase the adaptative ability of microorganisms from point of activation; this can occur either via mutation, recombination, or insertion, and it can enable the compound [144]. On the other hand, there are certain situations where although the cyanobacteria carry the intact gene cluster for Has, their production is not observed [81]. Some secondary metabolites are only generated in very specific situations or are present in minuscule quantities, making it difficult to detect [30,145].

Several *C. raciborskii* strains have also proven to be a potential source of Has. Despite the organizational similarity of their gene cluster in terms of Has production, each strain exhibits a peculiarity in the number and type of tailoring enzymes, thus indicating the biosynthesis of structurally diverse molecules [146]. The Hassallidin E gene cluster in PCC 8927 is the most divergent when compared with the orthologous gene clusters investigated in heterocystous cyanobacteria; these are the smallest gene clusters, comprising only 16 genes, and 13 are in concordance with the reference cluster of *Anabaena* sp. SYKE748. The existence of such gene clusters in this strain can be explained by a horizontal gene transference event as the region where the *Has* genes are situated is flanked by 48 genes; these are absent in the other 14 available *Planktothrix* genomes. Additionally, the GC content of this region considerably diverges from the whole genome [70]. 

**Figure 10:**
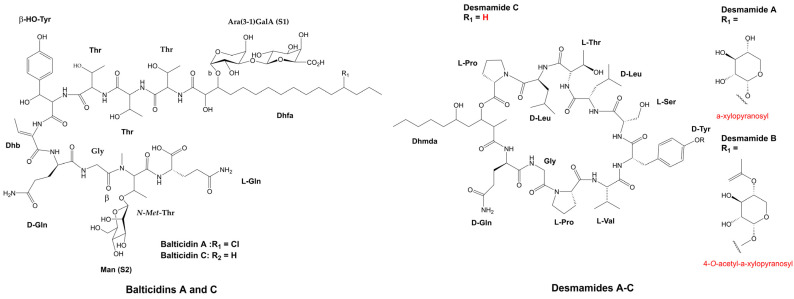
Chemical structure of Balticidin A and C, and the recently discovered Desmamides A–C.

The Has family also harbors the Balticidins (Figure 10), which were discovered in the Baltic Sea cyanobacterium, *A. cylindrica* Bio33. The Balticidins, as well as the Has, are glycosylated lipopeptides; however, they differ in some respects. The occurrence of a dihydroxyhexadecanoic acid side chain, connected to a disaccharide formed by arabinose and galacturonic acid, is found exclusively in Balticidins. Another feature that can be observed in some Balticidins, but is absent among Has, is the presence of chloride in the fatty acid residue. Balticidins B and D are analogs, and they possess circular structures, sharing the same core as Has A and B, differing only in that Thr is substituted with β-HO Tyr. In the disk diffusion assay, it was observed that Balticidins A–D (10 µg) display an inhibitory activity against *C. maltosa* SBUG700 (Table 2) [71]. 

Recently, a new glycosylated lipopeptides family named Desmamides was discovered in the plant facultative symbiotic cyanobacterium *D. muscorum* LEGE 12446 (Figure 10) [147]. The microorganism was isolated from the coralloid root of the cycad, *Cycas revoluta*. Despite some structural similarities with some lipoglycopeptides, the members of this group possess some particularities that distinguish them from any other family [147]. Different from the Has, the fatty acyl residue in Desmamides A–C forms part of the macrocycle. The connection is guaranteed due to an amide bond with a N-terminal amino acid, and an ester bond with a C-terminus in the peptide chain [147]. When in the Has, the sugar moiety occurs in the nonproteinogenic amino acid, *N-Methyl*-Threonine, and/or in the 3-hydroxy group of the fatty acyl moiety. O-glycosylation of Desmamides A–B occurs in the Tyrosine amino acid. Both compounds were tested against several Gram-positive and Gram-negative bacterial and pathogenic fungi at 100 μg mL^−1^. Only the plant pathogenic bacterium *Xanthomonas campestris* had its growth significantly affected, showing an IC_50_ value of 48 and 34 μM, respectively [147].

#### 3.1.4. Extracellular Peptides 

**Figure 11:**
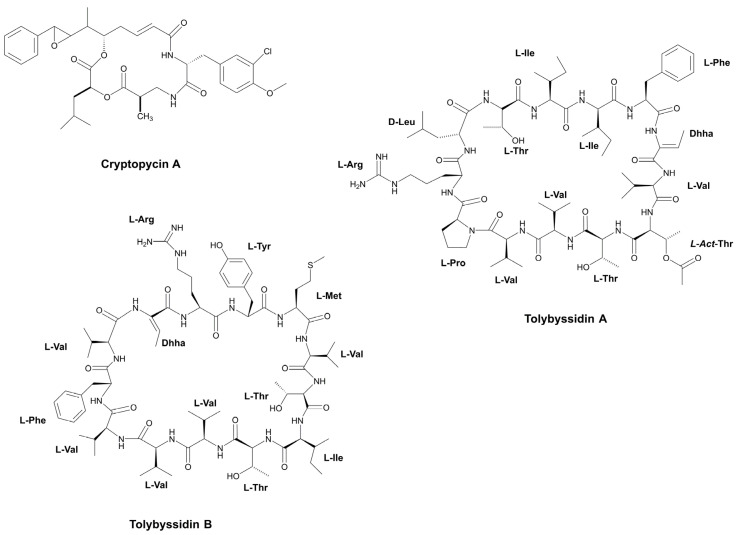
Example of fungicidal cyanopeptides released or excreted into the supernatant. Abbreviations: L-Arg (L-arginine), L-Tyr (L-tyrosine), L-Met (L-methionine), L-Val (L-valine), L-Thr (L-threonine), L-Ile (L-isoleucine), L-Phe (L-phenylalanine), Dhha (dehydrohomoalanine), L-Pro (L-proline), D-Leu (D-leucine), *O-Ac*-Thr (O-Acetyl-L-Threonine).

The fungicidal activity of oligopeptides found in the cyanobacterial exudate, has been investigated to a lesser extent. Typically, these compounds are referred to as allelochemicals and they have been associated with the defensive mechanism of cyanobacteria [148]. The cyclic tridecapeptides, Tolybyssidins A and B, belong to this category (Figure 11). Both are extracted from the culture medium of the cyanobacterium, *T. byssoidea* EAWAG 195 (Table 1). Moderate antifungal activity against *C. albicans* was observed in these metabolites, showing a MIC of 32 and 64 µg mL^−1^ (21.8 and 42.9 µM), respectively. The moiety, dehydrohomoalanine (Dhha), is present in both peptides, whereas D-amino acids are restricted to Tolybyssidin A [72]. Another peptide that is also obtained from a supernatant is the cyclic depsipeptide, Cryptophycin A (Figure 11). This peptide was first isolated from the marine sponge, *Dysidea arenaria*, and then identified as a product of cyanobacterial origin, as it was detected in the symbiotic cyanobacterium, *Nostoc* sp. GSV 224 (Table 1), which is currently known by the code, ATCC 53789 [149,150]. To date, over 28 analogs of Cryptophycin have been described, and all have been obtained exclusively from cyanobacteria belonging to the *Nostoc* genus [73,74,151,152,153,154,155,156,157]. The name Cryptophycin was conceived due to its ability to strongly inhibit the growth of various strains of *C. neoformans* (Table 2). The disk diffusion assay has demonstrated that in addition to *Cryptococcus* species, Cryptophycin A also inhibits 16 species of fungi (Table 2) [152]. Currently, metabolites belonging to this cyanopeptides family have mainly been obtained from cyanobacterial biomass. Due to their elevated cytotoxicity, the majority of investigations have focused on their application as chemotherapeutic agents in cancer treatment [73]. 

### 3.2. Phycobiliproteins

A protein group that exhibits antimicrobial activity is the phycobiliproteins group. These natural water-soluble pigments are mainly encountered in cyanobacteria, and the chloroplasts of algae belong to the *Rhodophyta phylum* [158]. They are the major pigments present in the antennae of these organisms, which are responsible for capturing light energy, and transferring it to the chlorophyll molecules. Phycobiliproteins can be classified, based on their spectral properties, into the following groups: phycoerythrin (red), phycocyanin (blue), and allophycocyanin (bluish-green) [159]. A considerable number of phycocyanins have demonstrated fungicidal properties against human and phytopathogenic fungi [160]. *Arthrospira* is one of the most investigated genera among cyanobacteria [161,162]. Purified phycocyanins in this group have demonstrated inhibitory properties against the fungi *A. niger*, *A. flavus*, *Penicillium* sp., *Rhizopus,* and *C. albicans* [161]. Their growth was also inhibited by the same type of pigment extracted from the thermotolerant cyanobacterium, *Synechocystis* sp. R10 [162] as well as those obtained from four *Tolypothrix* species isolated by Rao and coworkers [163]. The Phycocyanin of these filamentous cyanobacteria are also capable of impeding the growth of other fungi species such as *C. guilliermondii*, *A. niger,* and *A. fumigatus* without presenting a negative effect on Eri silkworm development [163]. 

The first phycoerythrin that originated from cyanobacteria with antifungal properties was reported in 2018 by Hemlata and colleagues [164] from the cyanobacterium, *Michrochaete*. At 200 µg mL^−1^, this peptide displays moderate and weak inhibition for *C. albicans* and *A. niger*, respectively. Antifungal phycoerythrin was also found in the strain, *Nostoc* sp. A5, which was obtained from a cliff face [160]. The high light intensity and low water availability of the isolation source of this cyanobacterium were associated with elevated phycoerythrin stability, and it maintained its antimicrobial properties for 10 days [160]. Recently, phycobiliproteins isolated from *Arthrospira platensis* presented antifungal properties for the phytopathogen *B. cinerea* [165]. This fungus occurs worldwide, it is capable of infecting various plant species, including those with high commercial value, and it can cause gray mold disease. In the presence of phycobiliproteins, this phytopathogen’s growth and sporulation were reduced compared with the control group. Furthermore, tomatoes previously treated with this pigment when artificially infected with *Botrytis cinerea* show a higher level of resistance [165].

### 3.3. Enzymes

An enormous variety of prokaryotic and eukaryotic organisms are well known for their ability to synthesize a considerable number of hydrolytic enzymes with antifungal properties [166,167,168]. In cyanobacteria, this feature has been poorly explored. There are few publications focusing on antifungal enzymes that originate from these microorganisms. The photosynthetic nature of cyanobacteria, which emit an organic carbon source, has contributed to differing views on the biocide potential of their enzymes. However, it is known that these living organisms employ diverse protective mechanisms against some protozoa, bacteria, and fungi, in which hydrolytic enzymes can be involved [26,169,170].

**Figure 12:**
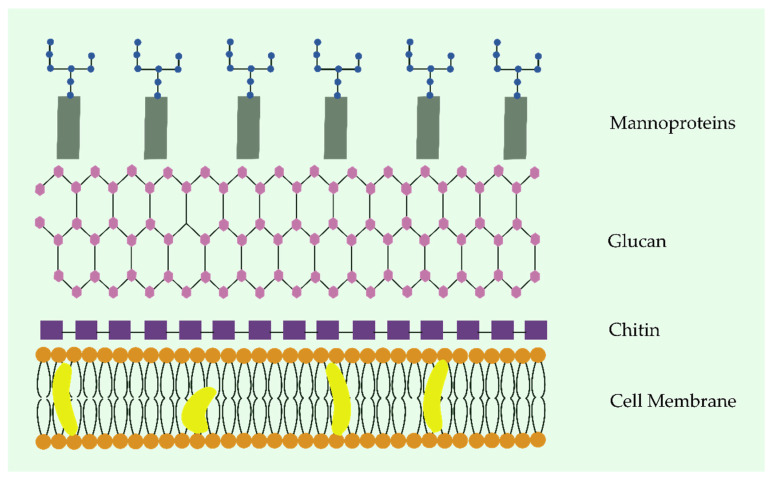
Fungal cell wall components. The fungal cell wall is composed of a membrane enriched with ergosterol (in yellow) and some proteins. It is also possible to observe the presence of a protective layer of chitin, as well as glucans, and mannoproteins on the surface.

The hydrolases primarily act in the fungus’s cell wall, which, in addition to being quite accessible, plays an important role in cell integrity. This barrier essentially consists of glycoproteins, glucans, and chitin, and in some situations, melanin is also observed [171,172]. The proteins of glycoproteins are covalently bound to carbohydrates either through their amide group, which is present in the side chain of asparagine, or through its oxygen atom, which is located in the side chain of threonine or serine [173]. They participate in various biologic processes, such as adhesion, protection against various types of molecules, and absorption of certain substances. Glucans are structural polysaccharides formed by glucose monomers that vary in terms of their glycosidic bond position, whereas chitins are polymers of N-acetylglucosamine, which are deposited nearby to the plasmatic membrane (Figure 12). All of these biomolecules have been investigated extensively as therapeutic targets [174]. 

Glucanases are the hydrolases responsible for breaking the fungal cell wall through the cleaving of glycosidic linkages. They have been reported in diverse organisms, including cyanobacteria [175,176,177]. An example is a terrestrial strain, *Calothrix elenkinii* RPC1, that produces a fungicidal β-1,4-endoglucanase belonging to the peptidase M20 superfamily. This enzyme exerts inhibitory activity against the oomycete, *Pythium aphanidermatum*, thus causing the disintegration of its mycelia. The open reading frame responsible for its production encodes a chain composed of 348 amino acid residues, which includes a signal peptide that reveals its extracellular nature. Different from endoglucanase, which is encountered in other aerobic microorganisms that possess a typical structure constructed by a mature protein with a catalytic domain bound to a cellulose-binding domain (CBD) through a peptide sequence rich in proline/threonine/serine, the endoglucanase secreted by *C. elenkinii* RPC1 contain neither the CBD nor the linker peptide sequence. Thus, it shows a higher similarity with endonucleases synthesized by invertebrates [178]. *A. laxa* RPAN 8 also produces fungicidal endonucleases that target *P. aphanidermatum*. Analyses performed by Gupta and colleagues revealed a mixture of two glucanases in this strain, one with 38 kDa (end 1), which exhibits β-1,4 endonuclease properties, and another with 73.89 kDa (end 2), whose activity was detected in both β-1,4 and β-1,3 endonuclease. Both enzymes exhibit tolerance to pH value variation, maintaining their activity for 12 h in a range of 5.0 to 7.0 for end1, and 5.0 to 9.0 for end 2. The tridimensional structure of their active site confirmed the presence of the thiol group in the catalytic site, and it demonstrated the importance of residues in a signal peptide with regard to antifungal activity [179,180]. 

**Figure 13:**
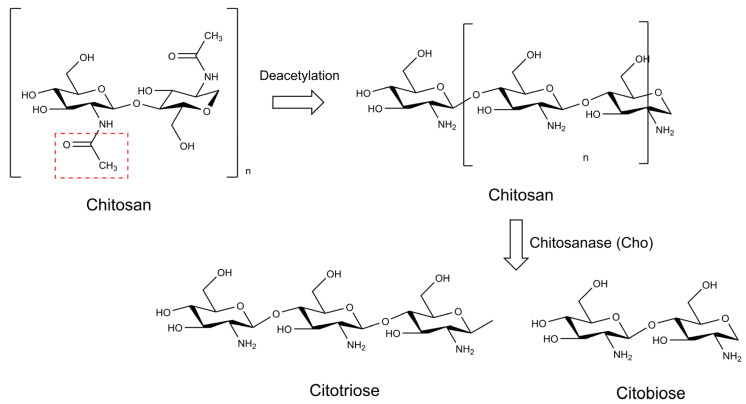
Hydrolysis of chitosan by the enzyme chitosanase (Cho), which is produced and released by the strain, *Anabaena fertilissima* RPAN1.

The chitinases are glycosyl hydrolases that degrade chitin. As this polymer is the second most abundant on the Earth’s surface, these enzymes fulfil a varied number of functions in nature [181,182]; for instance, they participate in the defense mechanisms of some plants and other organisms in response to infections caused by pathogenic fungi [183,184]. Chitin’s chemical degradation can lead to the production of its deacetylated form, the polysaccharide chitosan, which is further hydrolyzed by chitosanases (Figure 13). These hydrolases also assist in the digestion of the fungal cell wall [185]. In cyanobacteria, the occurrence of the homologs of chitosanases was initially reported in three strains of *Anabaena*: *A. laxa* RPAN8, *Anabaena iyengarii* RPAN9, and *A. fertilissima* RPAN1 [186,187]. Their production was positively correlated with the fungicidal properties observed in the supernatant of these microorganisms [186]. The chitosanase of *A. fertilissima* RPAN1 (Cho), in accordance with its catalytic site, is associated with the GH3-like family, and can degrade chitotetrose and longer chitosan oligosaccharides into dimers and trimers, but it does not significantly act on chitobiose and chitotriose. These data indicate the endo-type essence of Cho. Minor variations in its amino acid sequence, acquired from site-directed mutagenesis, reveal that Glu-121 and Glu-141 residues are crucial for their ability to restrict the growth of the fungus, *F. oxysporum*. Inhibition via glutathione, β-mercaptoethanol, dithiothreitol, silver, and mercuric cation suggests the involvement of a thiol group in the active catalytic site, whereas the enhanced activity after the addition of Cu^2+^ and Zn^2+^ indicates a possible interaction between these metals and two imidazole nitrogens obtained from histidine, which is a very common amino acid in chitosanases. The elevated pH levels and temperature stability of RPAN1 chitosanase makes it extremely promising when controlling phytopathogenic fungi in different soil types [188]. 

### 3.4. Carbohydrates and Their Derivatives

Polysaccharides exhibit enormous structural and functional diversity, which is mainly attributed to monomer composition, linkage types, ramification size, and connection with other chemical groups [189,190]. In cyanobacteria, they act in a very diversified manner, occupying the role of storage molecules, such as glycogen [191] and starch, and they integrate the cell envelopes in the form of lipopolysaccharides, which contribute to 70–75% of the surface of the cyanobacterial cell [192]. Some polysaccharides in cyanobacteria are secreted into the extracellular matrix, which is important for the defense of these microorganisms against toxins and predators. Furthermore, they can also act in communication with other microorganisms and during biofilm development [193,194]. An underexplored property of these polymers is their ability to inhibit bacteria and fungi.

Cyanobacterial polysaccharides may bear unusual features in comparison with other prokaryotes [195]. The terrestrial cyanobacteria, *N. commune* (Table 3), produces a polysaccharide that exhibits biocidal activity against the following microorganisms: *Bacillus subtilis*, *Escherichia coli,* and *A. niger*. The major constituents of this polymer are as follows: acyl-amino, hydroxyl, and pyranose. Its content can represent about 15% of *N. commune*’s biomass [83]. Belhaja and coworkers [81] identified a polysaccharide exhibiting inhibitory activity against *T. rhizoctonia* (MIC: 78 µg mL^−1^), *F. solani* (MIC: 39 µg mL^−1^), *F. oxysporum* (MIC: 78 µg mL^−1^), *A. niger* (MIC: 19 µg mL^−1^), and *C. albicans* (MIC: 78 µg mL^−1^) (Table 2) in the Tunisian *Phormidium versicolor* NCC 466 (Table 3). Chemical analysis revealed a mixture of different monomers, including arabinose, xylose, ribose, galactose, glucose, mannose, and rhamnose, among others. In addition to this heterogeneity, there was a greater amount of uronic acid and more sulfated groups, which are associated with the antimicrobial and antioxidant capacities of some polysaccharides [81,196]. 

The polysaccharide isolated from the strain, *Anabaena* sp. BEA 0300B, shows an inhibitory effect against the fungus *B. cinerea,* both in vitro and in vivo, using strawberries. The reduction in symptoms of the infected fruits was not only correlated with the direct action of the *Anabaena* sp. BEA 0300B polysaccharide on the phytopathogen, but also to its impact on the activation of the plant defense system, as it activated various signaling pathways [82]. Some authors have documented the ability of some algal polysaccharides to trigger plant defense mechanisms, as illustrated by the polysaccharide Laminarin, which is extracted and purified from the brown alga *Laminaria digitata*. At a dose of 200 µg mL^−1^, this polymeric carbohydrate induces hydrogen peroxide release in a few minutes in tobacco. After some hours, the cells of this plant exhibit an enhanced expression of the enzymes phenyl ammonia-lyase, caffeic acid O-methyltransferase, and lipoxygenase. All are somehow related to plant defense mechanisms [197].

Recently, Brilisauer and collaborators have examined antimetabolites in their search for an alternative to control the growth of plants and pathogenic microorganisms. These compounds are known for their ability to inhibit enzymatic activity by mimicking the enzyme substrate. The Brilisauer study discovered the uncommon sugar, 7-deoxy-sedoheptulose (7dSh), in the methanolic extract of a supernatant of a *S. elongatus* culture in the stationary phase. This antimetabolite acts as an inhibitor of the 3-dehydroquinate synthase, which is a crucial enzyme in the shikimate pathway. The enzymes belonging to this pathway cannot be substituted by any other alternative enzyme, and they are exclusively found in cyanobacteria, fungi, and plants; they are not present in mammal cells, which make them very promising targets. The end-products of the shikimate pathway are aromatic amino acids. The growth of *S. cerevisiae* is only affected by 7dSh when cultivated in the YNB minimal medium. In the YPD complex medium, the yeast growth was not altered. One plausible explanation for this difference is that the YPD medium offers an environment rich in nutrients and is therefore capable of reducing the deficiency of aromatic amino acids. A similar antifungal effect was observed for the herbicide glyphosate, but at a tenfold higher concentration than that documented for 7dSh [80,198].

### 3.5. Fatty Acids and Their Derivatives

**Figure 14:**
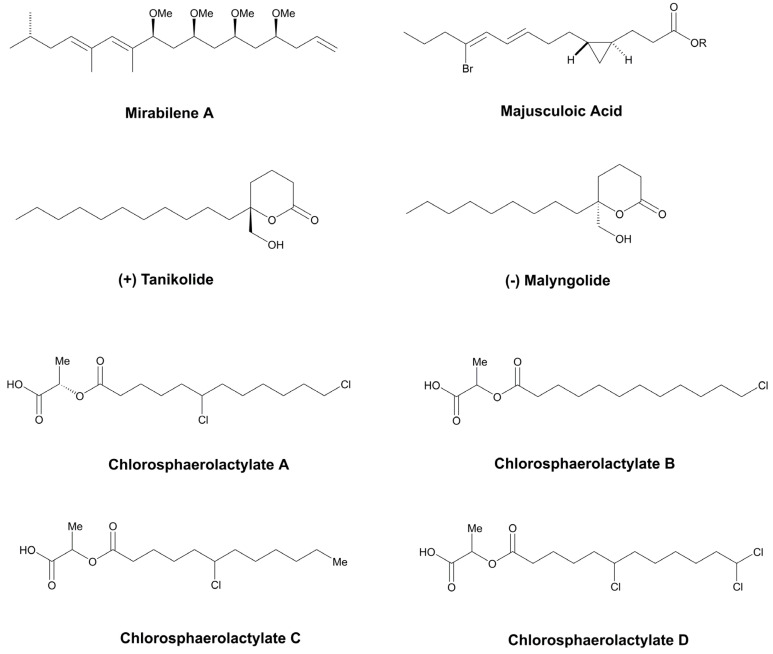
Fatty acids and their derivatives which possess fungicidal activity, isolated from cyanobacteria.

Cyanobacterial genomes harbor a suite of gene clusters that are associated with the incorporation of fatty acid-derived moieties [199]. This group of molecules can present diverse functions in the cell and they have mainly been investigated due to their biocidal properties and for the production of biofuel [200,201]. The first fatty acid derivatives with antifungal properties that were identified in cyanobacteria were the cytotoxic compounds, isonitrile mirabilenes A-F (Figure 14); these were obtained from the lipophilic extract of the cyanobacterium, *S. mirabile* BY-8-1 (Table 4). *A. oryzae* and *P. chrysogenum* had their growth weakly inhibited when exposed to this toxin [86]. 

Tanikolide (Figure 14) is another fatty acid derivative that exhibits antifungal activity. One hundred micrograms of this metabolite produced a zone of inhibition with a 13-mm diameter for *C. albicans*. It was first isolated from the lipophilic extract of the marine species, *L. majuscula*, as a result of its strong toxic effects against the brine shrimp, *Artemia salina*. Structurally, Tanikolide is composed of a lactone connected to a hydroxymethylene group, and an undecyl chain through the C-5 (Figure 14) [88]. One related compound is the Malyngolide (Figure 14), which is also formed by the same units, but its alkyl chain is slightly shorter and it has an opposite stereoconfiguration. In contrast to Tanikolide, Malyngolide (Figure 14) exhibits no anti-candidiasis activity; however, it exhibits anti-bacterial properties [88]. 

In addition to the lactone ring, modified fatty acids obtained from cyanobacteria, mainly those from the marine environment, can also contain some halogens, such as chlorine and bromine, in their structures. Such replacements can alter the biological activity of their natural products. MacMillan and Molinski (2005) [85] extracted a brominated cyclopropyl fatty acid from a marine cyanobacterial mat assemblage, called Majusculoic Acid (Figure 14). For the fungi, *C. albicans* ATCC 14503 and *C. glabrata*, this compound showed a MIC of 8 µM and 19.3 µM (Table 2), respectively. Moreover, it was incapable of limiting the growth of the fluconazole-resistant strain, *C. albicans* UCD-FR1 [85]. 

Recently, a novel series of esters of chlorinated lauric acid and lactic acid, called Chlorosphaerolactylates A−D (Figure 14), have been reported in the cyanobacterium, *Sphaerospermopsis* sp. LEGE 00249 [84]. All members of this group displayed weak antimicrobial and antibiofilm activity against multidrug-resistant clinical isolates, including the strain *C. parapsilosis SMI416* (Table 2). Chlorosphaerolactylates B–D possess the same molecular skeleton as Chlorosphaerolactylate A; they only differ in terms of position and/or the quantity of chlorine atoms linked to the lauryl moiety. The pathway responsible for their production has been reported by Abt et al., 2021 [202]. The structure of the Chlorosphaerolactylates resembles that of commercial lactylates, which are broadly employed as emulsifying agents in the cosmetic and food industries. Among the interesting features of this group of emulsifiers are their elevated biodegradability and low toxicity for humans.

### 3.6. Alkaloids 

Many bioactive molecules found in cyanobacteria belong to a group of alkaloids [28]. These compounds possess alkali-like properties and are structurally characterized by the presence of at least one nitrogen atom in a heterocyclic ring [203]. The genes responsible for their biosynthesis are widespread in nature and are found in higher plants and other microorganisms, as well as in many marine animals, amphibians, and arthropods [204]. Due to the presence of different active functional groups in the same molecule, most alkaloids can interact with multiple cellular components and can perform more than one biological function [28,205].

A variety of alkaloids belonging to the same family can be identified in a single organism, since such compounds are usually produced as a mixture of a few majors and various minors [28]. The fungicidal properties of many cyanobacterial extracts is attributed to the presence of certain alkaloids. The best-known representatives that possess this property are those belonging to the group of hapalindole-type alkaloids and Tjipanazoles, which were reviewed here, along with the individual compounds, Carriebowlinol, Norharmane, and Nostocarboline. 

#### 3.6.1. Hapalindole-Type Alkaloids

**Figure 15:**
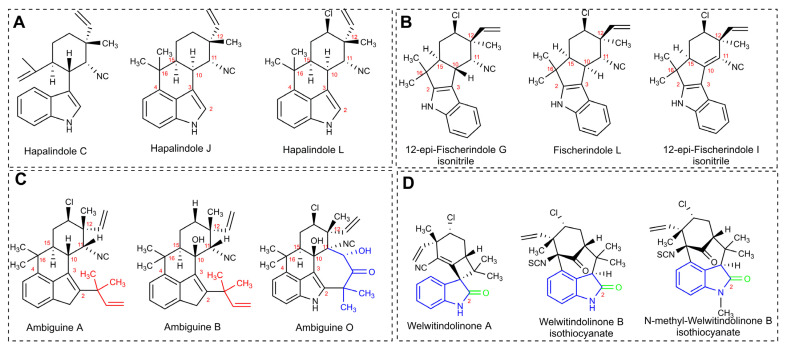
Hapalindole-type alkaloids. (**A**). Some examples of tricycle hapalindoles (Hapalindole C) and tetracyclo hapalindoles (J and C). In the tetracyclic hapalindoles, the key connection occurs between C-4 and C-16. (**B**). Chemical structure of some Fischerindoles, in which the key connection is between C-2 and C-16 (**C**). Chemical structure of Ambiguines A, B, and C. In red, the isoprene unit is attached to C-2, and in blue, the isoprenyl group is fused to the isonitrile-bearing carbon. (**D**) Some examples of welwitindoliones. In green, the oxidized carbon is in the second position, and in blue, the bicyclo is shown [4.3.1].

Hapalindole-like indole alkaloids belong to one of the largest groups of bioactive compounds originating from cyanobacteria (Figure 15) [206]. Members of this family have only been detected among those members of the Stigonematales order, and they are distributed into four classes: Ambiguines, Hapalindoles, Fischerindoles, and Welwitindoliones (Figure 15) [206,207,208]. The Stigonematalean cyanobacteria that are known for producing such natural products have been mainly isolated from freshwater and terrestrial environments. A single strain can produce various hapalindole-type alkaloids with remarkable structural diversity [209,210]. These metabolites are known due to their bioactivity against diverse organisms, including bacteria, insects, fungi, and cancer cells. The main differences between them concern the number and connection patterns of the rings, which are generated by variations in the chlorination, cyclization, and oxidation/reduction steps [92,209,211]. 

Hapalindole is the most predominant group, with approximately 31 members [206]. They normally exhibit a polycyclic system formed by four or three rings. These alkaloids were partially responsible for the antimicrobial activity of the lipophilic extract of the soil strain, *H. fontinalis* ATCC 39694 (Table 2) [89,95,212]. Moreover, similarly to Fischerindoles (tetracyclic), they possess stereocenters at C-10, C-11, C-12, and C-15, which guarantee a spectrum of stereoisomers. Both groups are also known for the incorporation of halogens and hydroxyl groups in their structure. The main difference between them is the key connection between the rings [206]. In Fischerindoles, the key connection is between the carbons located at positions 2 and 16, whereas in the tetracyclic Hapalindoles, this connection is between the carbons situated at positions 4 and 16 (Figure 15A,B) [206].

Ambiguines arose from the screening work of Smitka and coworkers using the terrestrial strains *F. ambigua* UTEX 1903, *H. hibernicus* BZ-3-1, and *W. prolifica* EN-3-1 against several fungi (Table 5). They differ from the Hapalindoles in that they have an additional isoprene unit attached to the C-2 of the indole moiety. Certain ambiguines have their isoprenyl group fused to the isonitrile-bearing carbon [89,90,92]. In 1998, Huber, Moore, and Patterson discovered ambiguine G in the cyanobacterium, *H. delicatulus* IC-13-1 (Table 5). This was the first documented ambiguine with a nitrile moiety, instead of the hallmark isonitrile [213]. In addition to their antimicrobial properties, ambiguines also target cancer cells and can suppress the root growth of lettuce [211,214]. The teratogenic effect has been also reported for the variant, 12-epi-ambiguine B nitrile, in zebrafish embryos [215]. 

The welwitindoliones are the most distinct hapalindole-like indole alkaloids, compared with the other groups. The defining feature of such compounds is the presence of an oxidized carbon in the second position and the bicyclo [4.3.1] decane ring system, which, with the exception of the welwitindolinone A isonitrile, is present in all other members [206,219,220]. Welwitindolinone A isonitrile and N-Methylwelwitindolinone were the first members of the group to be discovered in the terrestrial strain, *H. welwitschii* IC-52-3 (Table 5), and they were responsible for conferring fungicidal and larvicidal properties onto this strain, [99].

#### 3.6.2. Tjipanazoles

**Figure 16:**
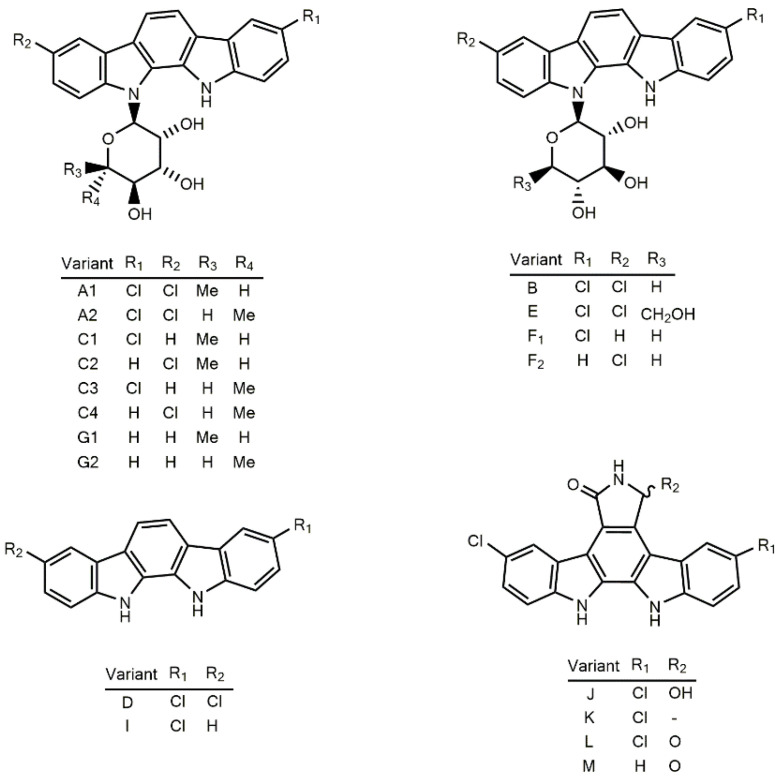
Chemical structure of members of the Tjipanazoles family.

Tjipanazoles (Tjipas) are polyhalogenated natural products belonging to the group of compounds possessing the indolo [2,3−a] carbazole pentacyclic system in their structure [98] (Figure 16). Members of this family have been explored as therapeutics due to their biological versatility. *T. tjipanasensis* DB-1-1 (Table 5) was the first identified source of these metabolites [98]. An investigation into the fungicidal properties of its lipophilic extract led to the obtainment of Tjipas Al, A2, B, Cl, C2, C3, C4, D, E, Fl, F2, Gl, G2, I. Furthermore, J. Tjipas Al and A2 display considerable fungicidal activity against rice blast and leaf rust wheat infections [98]; however, as with the other Tjipas, these compounds offer no in vivo protection against disseminated candidiasis in mice, and they possess a low level of toxicity when combatting leukemia and solid tumor cell lines [98].

Recently, Chilczuk and coworkers reported new Tjipas variants (K, L, and M), along with those known (D and I) in the terrestrial strain, *F. ambigua* 108b [221]. Tjipanazoles J, K, L, and M are the only ones in the class with the pyrrolo[3,4-c] ring. Such a moiety is present in analogous rebeccamycin, arcyriaflavin A, and K252-d. Similar to the variants J, K, L, and M, Tjipas D and I do not incorporate any sugar moiety into their structure [221]. Structural differences between these metabolites are related to their ability to inhibit the ABCG2 transporter (ATP-binding cassette superfamily G member 2). The pyrrolo [3,4-c] ring and chlorination are essential attributes in terms of biological activity [222]. The biosynthetic pathway of Tjipas has been elucidated, and it utilizes Two L-tryptophan residues to act as precursors. The transformation of these amino acids into Tjipanazoles D occurs through the actions of five enzymes (an L-tryptophan halogenase, an L-tryptophan oxidase, a chromopyrrolic acid synthase-like protein, a CYP 450 enzyme, and a FAD-binding monooxygenase) [222].

Recently, based on a polyphasic analysis involving morphological information and taxonomic data that were established from the study of 16S rRNA, a 16S–23S internal transcribed spacer (ITS), and the protein-coding gene *rbcLX*, *F. ambigua* 108b/CCAP1427/4/97.28 was reclassified as a member of the *Symphyonemataceae* family, receiving the name *Symphyonema bifilamentata* sp. nov. 97.28 [223]. Such information corroborates with the data obtained from a bioinformatic screening of homologous genes associated with Tjipanazoles biosynthesis across all genomes available in the NCBI database. *Fischerella* genomes are devoid of these genes [223].

#### 3.6.3. Individual Compounds

##### Carriebowlinol

**Figure 17:**
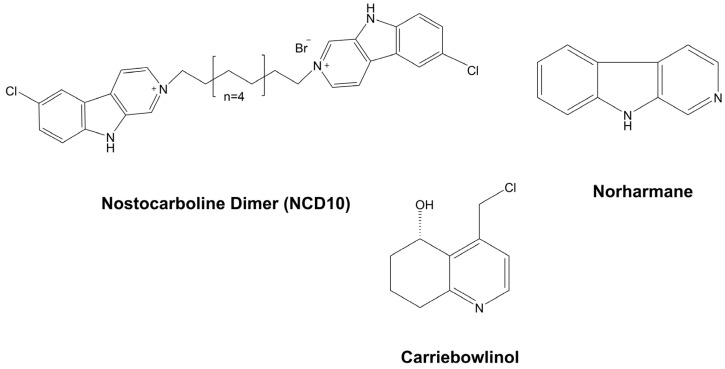
Chemical structure of alkaloids, with fungicidal properties, obtained from cyanobacteria.

Carriebowlinol is a quinoline alkaloid analog that is extracted from a cyanobacterial mat using Lyngbic acid; it is abundantly distributed on a coral reef located near Carrie Bow Cay in Belize (Figure 17). The components of this mat are two unidentified filamentous cyanobacteria. The most abundant species on this mat morphologically resembles *L. majuscula*, displaying thick and long filaments. A phylogenetic analysis of this microorganism revealed that it has an independent lineage whose evolutionary process is very dissimilar from any known cyanobacterial groups. The second biocomponent is closely related to those species of the genus *Hormoscilla*, which are frequently associated with other marine cyanobacteria. Due to the difficulty in separating one cyanobacterium from another, the exact source of the metabolite has not yet been determined. The antimicrobial potential of Carriebowlinol includes fungi of marine origin, including *L. thalassiae*, *D. salina,* and *Fusarium* sp., various bacteria belonging to the *Vibrio* genus, and the species *Paramoritella alkaliphila* and *Pseudoalteromonas mariniglutinosa*. The anti-proliferative properties of Lyngbic acid were demonstrated in all the aforementioned fungi, showing IC_50_ values that were tenfold higher than those obtained for Carriebowlinol (Table 2). The presence of both metabolites can justify the absence of microbial biofilm on the walls of these cyanobacteria [87].

##### Norharmane

Norharmane (9H-pyrido(3,4-b)indole) is known as the simplest example of β-Carboline. Norharmane (Figure 17) has not only been described in cyanobacteria, but also in various other organisms, such as some higher plants [224], algaecide bacteria [225], and in a few marine species, some dinoflagellates and sponges [226]. Its antimicrobial spectrum is ample, harboring bacteria, cyanobacteria, and fungi. Structurally, this alkaloid is formed by a pyridine ring fused to an indole skeleton. In 2006, Volk and Furkert were the first to document Norharmane production in the culture medium of a cyanobacterium (*N. harveyana*). The compound exerted a fungicidal effect on the growth of *C. albicans* ATCC 10231, with a MIC of 40 µg mL^−1^, and it behaved as an allelochemical which exhibited inhibitory action against cyanobacteria and pathogenic bacteria [97]. Norharmane is also one of the main constituents of the antifungal extracellular extract of *N. muscorum*, which exhibits a significant ability to control the proliferation of the fungus, *A. porri*, which is the etiological agent of the purple blotch disease [227]. The release of Norharmane into the environment has also been described for other cyanobacteria such as *S. aquatilis*, *A. cylindrica*, *A. inaequalis*, *A. siamensis*, *C. minutus*, *N. carneum*, *N. commune,* and *P. foveolarum* in concentrations that vary from 0.24 to 524.70 μg. L^−1^ [217,218].

##### Nostocarboline

Nostocarboline is a β-carboline alkaloid with an elevated similarity to Norharmane (Figure 17). It was primarily obtained from the aqueous methanolic extract of the freshwater cyanobacterium, *Nostoc* sp. 78-12A. Although this strain was initially highlighted by Flores and Wolk in 1996 as a propitious source for the acquisition of anticyanobiotics, the first work examining this alkaloid focuses mainly on its enzymatic inhibitory properties, which originally included acetylcholinesterase (IC_50_: 13.2 μM), and in a subsequent study, trypsin (IC_50_: 2.8 μM) [228,229].

Other attributes of Nostocarboline include its algaecidal properties, which exhibit inhibitory activities in the toxic cyanobacterium, *M. aeruginosa* PCC 7806, the nontoxic cyanobacterium, *Synechococcus* sp. PCC 6911, and the eukaryotic green alga, *Kirchneriella contorta* SAG 11.81, at a MIC of 1 µM [230,231]. Interestingly, the metabolite also has a negative impact on the growth of the producing strain, but at a much higher concentration. This significant difference in toxicity can offer an ecological advantage through the release of the compound [232].

Based on the ability of Nostocarboline to target the photosynthetic apparatus, its deleterious effect has been extended to some protozoans; this is because some of these eukaryotic organisms contain apicoplast, which is one plastid-like organelle that is inherited from a secondary endosymbiotic relationship with an alga [233]. At a nanomolar concentration, the compound exhibits a specificity for *Plasmodium falciparum* K1, and it is inactive when used with *Trypanosoma brucei rhodesiense* STIB 900, *Trypanosoma cruzi* Tulahuen C2C4, and *Leishmania donovani* MHOMET-67/L82. The recorded IC_50_ value for the L6 rat myoblast cell line is nearly 620-fold higher than that observed for Plasmodium falciparum K1, therefore demonstrating considerable selectivity [234].

Although the bioactivity of this anticyanobiotic that works against fungi and bacteria is modest or absent, all ten dimers constructed from this metabolite demonstrate antimicrobial properties. Dimers with larger linker chains exhibit a better antagonistic activity. Among these, only the largest linking chain presented an inhibitory action against the yeasts, *S. cerevisiae* A-136 and *C. albicans* T-3419 (Table 2) [96].

### 3.7. Polyketides

**Figure 18:**
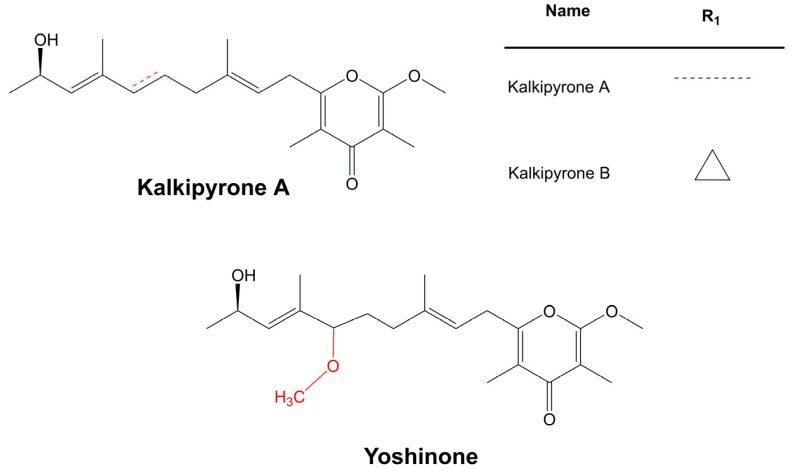
Chemical structure of the Polyketides, Kalkipyrones A–B, and Yoshinone. In red, the difference between Kalkipyrone A and Yoshinone is shown.

Despite the enormous diversity in identified polyketides, there are few antifungal polyketides in cyanobacteria. The extract of the cyanobacterium, *Leptolyngbya* sp. (Table 6), from the screening assay was pinpointed as being a promising source of antineoplastic agents. Further purification steps of this material led to the isolation of two pyrone-containing polyketides: Kalkipyrones (Kalkis) A and B (Figure 18). They differ only in the presence of an unsaturated bond between C-10 and C-11 in the alkyl chain. Such a difference does not seem to determine how toxic they are to the fungus, *S. cerevisiae* ABC16-Monster, since both exhibit a moderate activity against this microorganism, with IC_50_ values that are very close together (14.6 and 13.4 µM, respectively). In contrast, their bioactivity against H-460 human lung cancer cells showed a considerable difference in bioactivity. The unsaturated bond led to a 10-fold decrease in activity [101]. Kalki A had also been detected in an assemblage of the marine *L. majuscula* and *Tolypothrix* sp., obtained from a splash zone. It demonstrated elevated toxicity against *Artemia salina* (LD_50_ 1 µg mL^−1^), and ichthyotoxicity against goldfish (LD_50_ 2 µg mL^−1^). Attempts to determine which associated species were the source of this polyketide were inconclusive [235].

Another polyketide with antifungal properties that work against *S. cerevisiae* was isolated from an environmental sample containing the cyanobacterium, *Schizothrix* sp. The compound is known as Yoshinone A, and it has been detected in other organisms (Figure 18) [236]. Its structure bears various resemblances to Kalki A. The only difference between them is the occurrence of a methoxy subunit at C-11 of Yoshinone A, which is absent in Kalki A. The presence of this additional subunit was determined as being the main cause of lower bioactivity in Yoshinone A compared with Kalkis [101].

**Figure 19:**
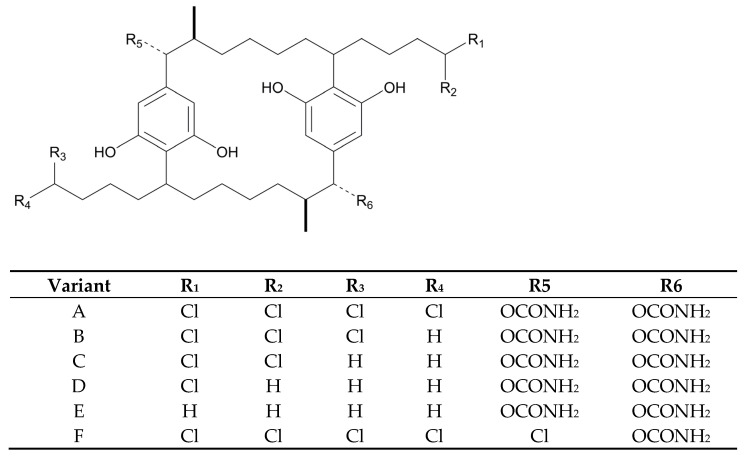
Carbamidocyclophanes’ (A–F) chemical structures.

Cyclophanes are distinct polyketides formed by an aromatic ring system that is connected with alkyl chains. The rigidity of these compounds can be attributed to the presence of rings, whereas their flexibility is determined by aliphatic units [237]. Carbamidocyclophanes (Carbami) are cyclophanes produced by certain cyanobacteria, and they exhibit strong cytotoxic activity against cancer cell lines. Additionally, they exhibit antimicrobial potential, mainly for gram-positive bacteria (Figure 19) [238,239]. Their fungicidal properties have not been well-emphasized. Among the Carbamidocyclophanes identified, only Carbamidocyclophanes A, B, and F have demonstrated appreciable anti-*Candida* activity, presenting MIC values of 5.5, 1.3, and 2.9 µM (Table 2), respectively [102]. Freshwater and terrestrial species belonging to the genera, *Cylindrospermum* and *Nostoc*, have been the exclusive sources of these substances (Table 7) [240,241].

### 3.8. Macrolides

**Figure 20:**
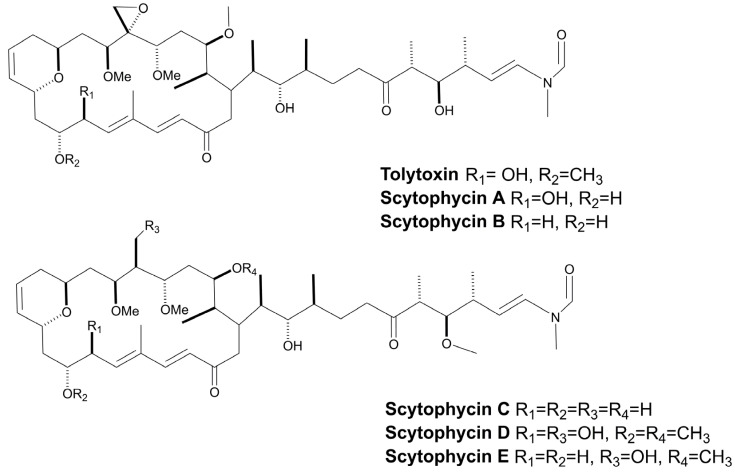
Chemical structure of Scytophycins.

Several macrolides are responsible for the fungicidal properties of a considerable number of cyanobacterial extracts. Scytophycins (Scytos) and Tolytoxin are the best-known antifungal and cytotoxic macrolides associated with these photosynthetic microorganisms (Figure 20) [243]. Scytos A and B (Figure 20) were two of the first members of this family to be documented in the terrestrial strain, *S. pseudohofmanni* ATCC 53141 [103]. Scyto B exhibits elevated toxicity levels in mice, with a minimum lethal dose value of 650 µg/kg, and similarly to Scyto B, it displays moderate activity against distinct types of cancer cell lines, both in vivo and in vitro. The antifungal properties of both, as well as of variants of Scytos C–E, were demonstrated for the fungi *S. pastorianus*, *N. crassa*, *C. albicans*, *P. ultimum*, *R. solani,* and *S. homoeocarpa* (Table 2) [103]. Treatment of Scyto B with acetic acid and ethanol leads to the formation of an aldehyde, which, once reduced with sodium borohydride, produces a primary alcohol. Both derivatives (the aldehyde and the primary alcohol) also exhibit antifungal properties [244].

Scytos are patented as a fungicidal solution that will enable the control of phytopathogens, either through their application in soil or directly on the plant’s surface [245]. Other cyanobacteria have demonstrated the ability to synthesize these macrolides. From an antifungal screening, comprising a total of 194 cyanobacterial strains isolated from distinct environments in Finland, Brazil, the United States, Australia, the Czech Republic, Sweden, Switzerland, Denmark, and Bermuda, two Scytos producing *Anabaena* strains were detected: *Anabaena* sp. HAN21/1, collected from a lake in Denmark, and *Anabaena* cf. *cylindrica* PH133, obtained from an aquatic gastropod. The same study also observed Scytos production in the benthic strains, *Scytonema* sp. HAN3/2 and *Nostoc* sp. HAN11/1 [14]. *C. muscicola* and *Nostoc* sp. 5/96 are the other two sources of these fungicidal metabolites, which have the ability to produce an enormous diversity of variants [246,247]. The main difference among the members of the Scytos family concerns the positions C-6, -7, -16, -19, -23, and -27 [14].

Tolytoxin A (Figure 20) belongs to the Scytos group, and it is described as 6-hydroxy-7-O-methyl-scytophycin B. The name refers to the first cyanobacterium that was identified as a producer of this substance *(T. conglutinate*). Its structure is very similar to Scytophycin A and B [244]. Its antifungal potential is comparable to that of the commercial antimycotic, Nystatin, exhibiting MICs in the ranges of 0.25 to 8 nM, which works against yeasts and filamentous fungi. Regarding its antibacterial potential, this Scyto fails to produce any type of activity that works against various tested bacteria at a 1 nM concentration [104].

**Figure 21:**
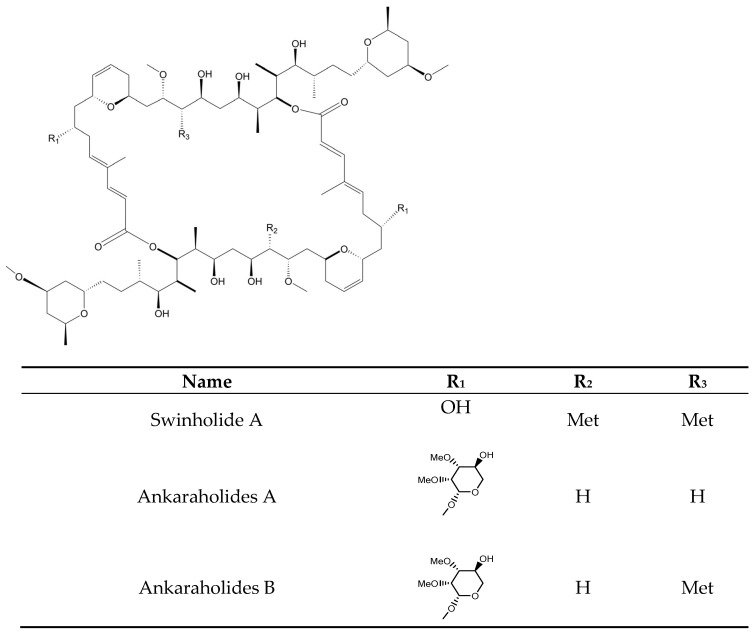
Chemical structure of the macrolides Swinholide A and Ankaraholides A–B.

A 42-carbon ring macrolide, known as Swinholide A (Swin A) (Figure 21), which shares significant similarities with Scytos, was identified in a cyanobacterium that also belongs to the genus, *Scytonema* (Figure 21) [248]. Swinholide A (Swin A) was first detected in the Red Sea sponge, *Theonella swinhoei*, in 1985, by Kashman and Carmely [105]. Its absolute stereostructure was only determined five years later [249]. It was first documented as a monomer, but it was later described as a dimer [249]. In cyanobacteria, its only occurrence was observed in 2005 by Andrianasolo and coworkers. These authors detected two additional glycosylated variants of (Swin A) in the cyanobacterium, *Geitlerinema* sp., which were isolated from the Nosy Mitsio Island; they were given the name Ankaraholides (Ankara) A and B (Figure 21) [248]. Ankara A and B exhibit the same structure, differing uniquely in that there is an additional methyl group linked at C-16, which is positioned in the sugar moiety (Figure 21). Ankara A displays strong cytotoxicity toward a variety of tumor cells, with IC_50_ values ranging from 8.9 nM to 262 nM. Recently, the same research group documented a series of Swinholide-related compounds, called samholides, using an environmental sample containing the cyanobacterium, *Phormidium* sp. They diverge as the methylation and esterification patterns present in the sugars, and via double-bond geometry. All nine Samholides that were isolated from the sample were cytotoxic to the H-460 human lung carcinoma cell line at nanomolar concentrations [250], except for Swin A, whose antifungal activity was detected, but scarcely explored; none of the compounds that were related to this macrolide and isolated from cyanobacteria had their antimicrobial properties investigated. However, these molecules are very promising with regard to antimicrobial properties, since they exhibit elevated levels of cytotoxicity and are extremely similar to Scyto family members [105,248].

**Figure 22:**
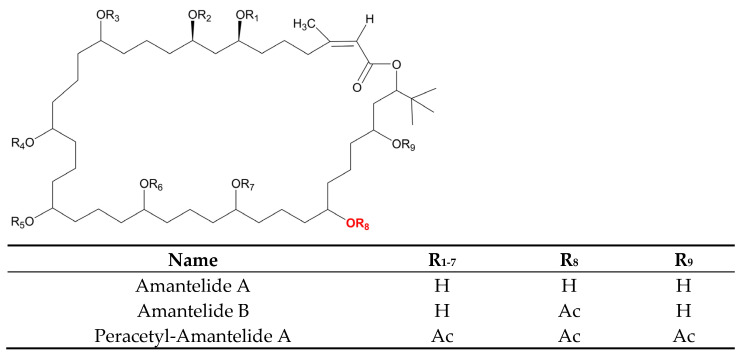
Chemical structure of Amantelides A–B and the Peracetyl-Amantelide A.

Amantelides A and B are polyhydroxylated macrolides extracted from the biomass of a Guamanian marine cyanobacterium belonging to the *Oscillatoriales* family (Figure 22). The broad-spectrum activity of Amantelide A includes cytotoxicity toward HT29 and HeLa cancer cell lines. It also includes antimicrobial activity against the marine fungi *D. salina*, *L. thalassiae*, and *Fusarium* sp. (Table 2), the gram-positive bacterium *S. aureus*, and the gram-negative bacterium *Pseudomonas aeruginosa* at submicromolar concentrations. A comparison of its activity with Amantelide B and Peracetyl-Amantelide A suggests that the hydroxyl group at C-33 plays an important role in enhancing its cytotoxic abilities. Monoacetylation at this position, as visualized in Amantelide B, results in an increase in IC_50_ values by 14- and 11-fold for HT29 and HeLa cells, respectively [106]. The consequences of these modifications on antifungal properties were demonstrated to be more severe for the fungi *S. cerevisiae* and *Schizosaccharomyces pombe*, wherein the compound activity was totally abolished [251].

**Figure 23:**
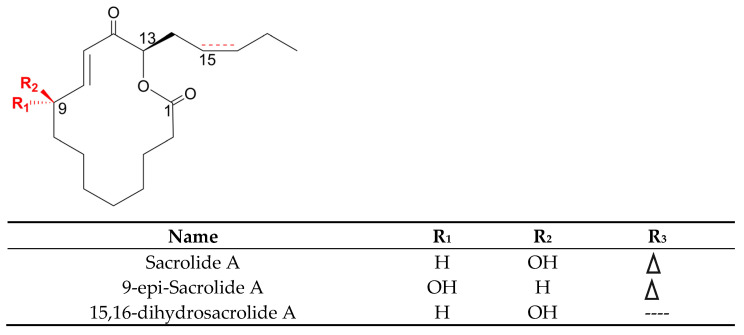
Structure of Sacrolide A and its congeners 9-epo-Sacrolide A and 15,16-dihydrosacrolide A.

Structure−activity relationship studies have also been applied to the macrolide, Sacrolide A (Sacro A), a member of the oxylipins, obtained from the ethanolic extract of the freshwater cyanobacterium, *Aphanothece sacrum* (Table 8), along with its congeners 9-epi-sacrolide A and 15,16-dihydrosacrolide A (Figure 23) [107,108]. Sacro A possesses antimicrobial properties that work against the gram-positive *Bacillus subtilis*, *Micrococcus luteus*, *Staphylococcus aureus*, *Streptomyces lividans*, and the fungi *C. albicans*, *S. cerevisiae,* and *P. chrysogenum* (Table 2). This metabolite displays superior toxicity against the fungus *P. chrysogenum*, and the bacteria *S. aureus* and *S. lividans* in its variants. Compared with its congener 15,16-dihydrosacrolide, Sacrolide A shows an eightfold improvement in its activity against *P. chrysogenum*. The variant 15,16-dihydrosacrolide differs from Sacro A only because of the presence of an unsaturated bond at C-15 in the aliphatic chain, whereas the 9-epi-sacrolide A differs due to an epimerization of C-9. The latter does not exhibit bioactivity against any of the abovementioned microorganisms. This finding suggests that the hydroxyl group at this position also exerts a crucial role in toxicity modulation [107,108].

### 3.9. Phenolic Compounds

**Figure 24:**
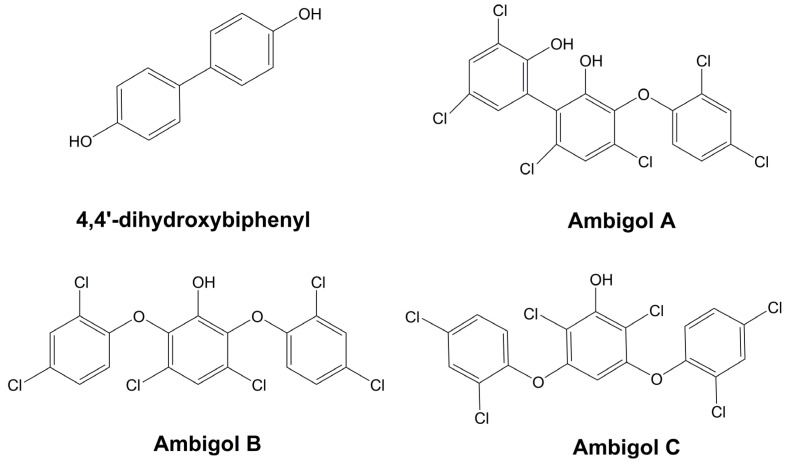
Phenolic compounds with antifungal activity of cyanobacterial origin.

Phenolic compounds are widely distributed in nature, occupying a remarkable place in terms of diversity and abundance. Some works have associated the antiproliferative properties of cyanobacterial extract with the high relative quantity of these antioxidant compounds [253,254,255]. They are normally produced in response to a stress condition or as a defense mechanism against pathogens and competitors [256]. The cyanobacterium, *Nostoc* sp. 54.79, releases the polyphenolic metabolite 4,4′-dihydroxybiphenyl into the environment (Figure 24). The exometabolite has algaecidal and antifungal effects, thus exerting an inhibitory activity on the growth of the yeast, *C. albicans*, with a MIC of 32 µg mL^−1^ (171.8 µM) (Table 2). Phenol compounds with antagonistic properties against fungi and bacteria have also been detected in the supernatants and biomasses of two distinct strains of *Nostoc muscorum* [253,257].

Ambigols (Ambi) (Figure 24) fail in the class of phenolic compounds with fungicidal properties. This family was first found in the terrestrial cyanobacterium, *S. bifilamentata* sp. nov. 97.28. It was found to be bioactive for *Bacillus subtilis*, *Biomphalaria glabrata*, and *P. oxalicum*. Some members of this family can also stop the action of the cyclooxygenase and HIV reverse transcriptase enzymes from performing their required actions [258]. Structurally, they resemble the polyhydroxylated phenol, Vidalol A, and some Phlorotannins, which are characteristic metabolites of the red algae, *Vidalia obtusiloba*, and certain brown algae, respectively. In contrast to such algal metabolites, Ambi are formed by three aromatic rings with multiple chlorine substituents [258,259]. Both Ambigols A and C reveal selectivity as their bacterial target, and they do not enforce any significant impact on the growth of gram-negative bacteria. The former, as compared with Ambigols B–C, is more interesting in biotechnological and ecological terms, since it exerts considerable deleterious effects on a variety of living beings, including the filamentous fungi *M. violaceum*, *E. repens*, *F. oxysporum*, and *M. microspora.* Of these, only *M. microspora* was also inhibited by Ambigol C [109,260]. In 2020, two further Ambis were documented (Ambis D–E), and their inhibitory activity was confirmed in *Serratia* sp. ATCC 39006. The biosynthetic pathway of Ambigols has recently been elucidated, and it involves enzymes belonging to the shikimate pathway, which is responsible for aromatic amino acid production [261,262].

### 3.10. Other

**Figure 25:**
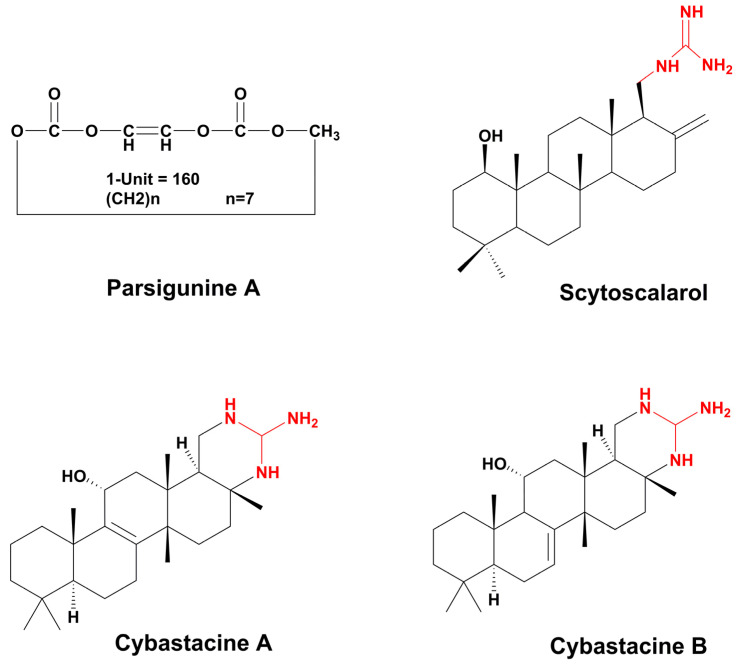
Structure of the polymer Parsiguine A, terpenoid Scytoscalarol, and Cybastacines A–B. In red, the guanidine group is shown.

An unrelated metabolite is the cyclic polymer, Parsiguine (Figure 25), which can be isolated and identified from the supernatant of the terrestrial strain, *Fischerella ambigua* PCC 1635 (Table 9). At least 20 µg mL^−1^ of this substance is required to inhibit the growth of the fungus, *C. krusei* ATCC 44507 [110]. Another polymer of a non-identified nature was extracted from the spent culture medium of a cyanobacterium from the genus, *Nostoc*. It exerted a negative effect on the development of the fungi *C. gloeosporioides*, *Fusarium verticillioides*, *B. cinerea,* and *Fusarium* sp. The polymer was applied to the production of a biofilm with very promising features for the food industry once it was capable of providing protection against some microorganisms, and when it had improved the nutritional value of the foodstuff [263]. Recently, notable attention has been paid to terpenoids that are extracted from cyanobacteria due to their enormous biotechnological potential [264]. The majority of studies focusing on the antimicrobial properties of these molecules have mainly focused on their antibacterial properties, with a scarce number of investigations focusing on their antimycotic potential [19]. Scytoscalarol (Figure 25) was the first sesterterpene documented in cyanobacteria and the first sesterterpene with a guanidine moiety isolated from a natural source. This was achieved via an antimicrobial screening that used the crude extract of the terrestrial strain, *Scytonema* sp. UTEX 1163. The bioactivity of Scytoscalarol was demonstrated for *B. anthracis*, *S. aureus*, *Escherichia coli*, *C. albicans*, and *M. tuberculosis*, showing MIC values in the range of 2 to 110 μM [111]. Recently, two other antimicrobial sesterterpenes bearing a guanidino group were extracted from the strain, *Nostoc* sp. BEA-0956, which was isolated from the wall of a cave located in Montañón Negro (Spain). The compounds have received the names Cybastacines A and B (Figure 25), and they have demonstrated moderate antibiotic activity. The guanidine group is considered to play a crucial role in the biological activity of both compounds since the hydrogen atoms present in its structure must facilitate the communication between molecules and the target protein. Furthermore, the protonated form of this residue, which is predominantly found at the physiological pH, seems to favor the interaction between antibiotics and the plasmatic membrane of bacteria [265].

## 4. Major Targets

### 4.1. Cell Membrane

**Figure 26:**
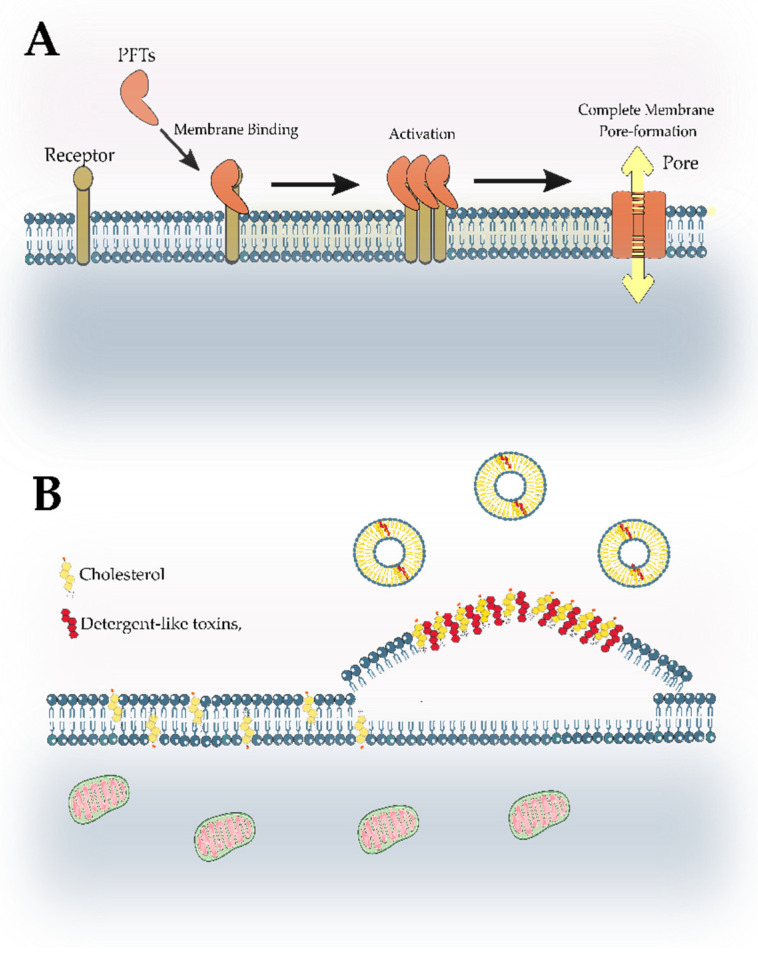
Molecular mechanisms of membrane-targeting compounds. (**A**) Pathways of pore-forming toxins (PFTs). Soluble PFTs move to plasmatic membrane and bind to receptors molecules. Then, they oligomerize on the surface of the membrane and produce transmembrane pores. (**B**) General mechanism of detergent-like toxins. These molecules normally bind to the external monolayer of the plasmatic membrane containing cholesterol and promote the vesicles formation and lateral phase separation.

The cell membrane of mammalians and fungi exhibits a high degree of similarity; they differ mainly in terms of lipid composition. Mammalian cells have a cell membrane composed of cholesterol whereas fungal cells have a membrane enriched with ergosterol. Both play similar functional and structural roles, acting on the fluidity and integrity of the membrane [266,267]. Such a contrast between the cells makes ergosterol a very promising target. The metabolites originated from cyanobacteria, and they have the ability to perturb the membranes of organisms. Their action mechanism has been elucidated, principally by employing mammalian cells. One of the main features of these compounds is their rapid cytotoxic effect, which can induce cell death within minutes [268]. Some act as a detergent, solubilizing the lipid component, whereas others induce pore formation (Figure 26). Abls A and B are inserted into the former group, specifically affecting those membranes with a significant quantity of cholesterol. Consequently, they are incapable of targeting the mitochondrial membrane, even at concentrations superior to those necessary to provoke cell lysis since such a membrane approximately contains only 5% cholesterol. The Abls activity spectrum also extends to anucleate cells, as erythrocytes [269]. During the rupturing of these cells, morphological transformations occur, wherein both discocytes and echinocytes form, accompanied by the release of adenine nucleotide and hemoglobin. The behavior of Abls is similar to that of the detergent, digitonin, which also possesses an amphipathic nature and has a neutral polar head at a physiological pH. The absence of a positive charge seems to be related to cholesterol dependency [269]. Curiously, at sub-lytic concentrations, Abls facilitate the entrance of various compounds without causing any type of injury at the membrane level. One example is the toxin nodularin, which is not internalized by kidney epithelial cells that are isolated from normal rats (NRK); however, when combined with Abl A, its entry is facilitated, which therefore leads to cell apoptosis. The absorption of plasmids that encode green fluorescent proteins into human embryonic kidney 293 cells (HEK 293) is also promoted by the activity of Abl A [269].

Sterol dependence may explain the selectivity of some compounds for eukaryotic cells, as noted in Has, which are not capable of altering the bacterial growth, but are nevertheless known for inducing cell death in both normal and malignant cells with very similar EC_50_ values [270]. These peptides are required in a slightly higher concentrations for the disruption of ergosterol liposomes, compared with cholesterol liposomes. Combined with the presence of a cell wall composed of chitin, this may explain the higher EC_50_ value recorded for *C. albicans*. Despite the fact that molecules of sterols do not directly interact with Has, they are responsible for offering a better organized and structured membrane for peptide insertion and conformational stability [270]. The first contact that Has has with the cell membrane occurs through its amino acids. The lipid tail is only introduced into the cholesterol shortly afterwards. Once inserted into the plasma membrane, the polar head of the Has molecules produces a conical geometry that modifies the membrane curvature to a more convex form, thus leading to its disruption [270].

The plasmatic membrane is also the target of the lipopeptide, Musco A. At a dosage of 25 μM, this undecapeptide rapidly increases the outer cell membrane’s permeability, thus leading to the entrance of small ions, and consequently, increasing the Ca^2+^ concentration inside the cell. At a temperature of 25 °C, its action on the cell membrane can occur in the absence of non-phospholipidic components, such as sugars and proteins; however, at 37 °C, the toxicity of the compound is limited to the presence of cholesterol and sphingomyelin [39]. The suggested mechanism of action is associated with its ability to diminish the fluidity of the cell membrane, whereas the structural abnormalities in the mitochondria are allied with the significant quantity of Ca^2+^ in the cytoplasm. Curiously, the membrane of this organelle, and the cytoskeleton of the cell remain intact during exposure [39]. Similarly to Muscotoxin A, the Puwas F and G are also responsible for provoking a strong and sudden increase in the intracellular concentration of calcium after causing damage to the plasmatic membrane. However, different to Muscotoxin A, these cyclic decapeptides alter the cytoskeletal structures, thus promoting the translocation of the cortical actin to an unusual location—the nuclear envelope. This phenomenon is unique and extremely specific, requiring more advanced studies for better elucidation [271]. The cytotoxic effect of Puwa F has also been demonstrated in human colorectal adenocarcinoma caco-2 cells through the determination of the total protein concentration of the cell’s lysate and lactate dehydrogenase enzymes. A model of the intestinal barrier built from these cells, as well as an evaluation of the expression of tight junction proteins, revealed that although Puwa F in non-cytotoxic concentrations is not capable of penetrating the monolayer, it can cause significant dysfunction from a biological point of view, as it increases the production levels of the inflammatory cytokine, interleukin 8 [272].

Rat fibroblastic cells treated with Sacro A at a dosage of 13 μM rapidly loses its ability to bind to the surface. This event is followed by blebs formation on the cellular surface. The anchorage loss caused cell death. Since these alterations occurred in a very short period of time, and as the cell does not initiate the cell cycle, procedures linked to DNA, RNA, and protein synthesis does not seem to be the target of this macrolide. Despite some phosphatase inhibitors of a lipophilic nature being known by their ability to form pronounced protuberances on the cell surface, this is not the mechanism employed here; this is because this enzyme is not inactivated by Sacrolide A [108].

### 4.2. Cytoskeleton

Many antifungal metabolites extracted from cyanobacteria can cause damage to one or more cytoskeleton components. The cytoskeleton is one of the main structures responsible for the maintenance of the shape and internal organization of cells [76,273,274]. Its major constituents are the microtubules, actin filaments, and intermediate filaments. As the name suggests, microtubules possess a tubular structure that is formed from a combination of 13 protofilaments composed of polymers of αβ-tubulin heterodimers. The side on the cell’s periphery is called the plus end since it grows faster than the opposite side; the opposite side is known as the minus end, which is anchored at the centrosome-containing microtubule-organizing center (MTOC), near the cell nucleus [275,276]. The microtubule’s dynamism is guaranteed by a diverse range of factors. Among them are GTP hydrolysis using β-tubulin, the production of different tubulin isotypes, as well as post-translational modifications. Such microtubule movements play a crucial role in chromosome migration during mitosis through their interaction with the kinetochores of chromosomes [277].

**Figure 27:**
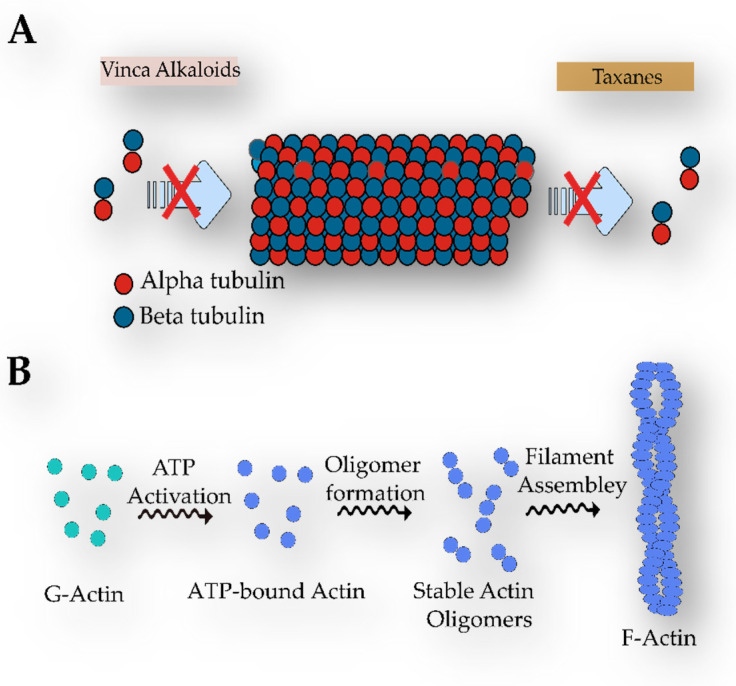
Structure of a Microtubule and the Actin Filaments. (**A**) Mechanism of action of vinca alkaloids and taxanes. Although taxanes promote microtubule stabilization by avoiding the release of dimers of tubulin, vinca alkaloids block microtubule polymerization. (**B**) F-actin formation.

Microtubule-targeting agents have not only been researched due to their fungicidal potential, but also (and mainly) because of their anticancer properties. Many of these metabolites have received FDA approval and are all-natural products; alternatively, they have been derived from compounds initially identified in nature [278]. The diversity of mechanisms employed by such compounds allowed their classification into two groups: microtubule destabilizers or stabilizers (Figure 27A). Molecules belonging to the former group provoke microtubule depolymerization, and consequently, they diminish the density of the material within the cell. In the second group, agents capable of intensifying tubulin polymerization and increasing its density within the cell are present, thus resulting in the formation of intracellular microtubule bundles [279]. Several compounds of diverse structures and origins are known as they promote the stabilization of microtubules. These agents include taxanes, epothilones, and the polyketide, discodermolide. Agents that destabilize microtubules consist of vinblastine, colchicine, vinca alkaloids, dolastatins, halichondrin B, and cryptophycins [280]. One main difference between them lies in the tubulin-binding sites. Currently, four sites have been identified for microtubule depolymerizers, namely, colchicine, vinca, maytansine, and the pironetin domain [278]. Cryptophycins bind at a site that may overlap with the vinca binding site [281]. This finding is based on the study conducted by Mooberry, Taoka, and Busquets in 1996 [282] They evaluated the changes in the tubulin structure using a proteolysis pattern with trypsin and chymotrypsin. The latter enzyme, hydrolase β-tubulin at tyrosine 281, formed an amino-terminal sequence with an apparent mass of 34 kDa, and a carboxyl-terminal portion with an apparent mass of 21 kDa. Colchicine exposure prevents the construction of such segments through the induction of a local unfolding around arginine 390 in the carboxyl terminus. This change alters the cleavage site of chymotrypsin to phenylalanine 389, and consequently, it leads to the formation of a segment with an apparent molecular mass of 43 kDa, which is designated as β-Col, and it is a minor cleavage product. Different from Colchicine, and similar to the vinca alkaloid vinblastine, Cryptophycin 1 causes opposite effects. Its contact with β-tubulin inhibits the production of the minor tryptic cleavage product, and it results in the non-detection of β-Col. Another shared feature between vinblastine and Cryptophycin 1, as well as other vinca alkaloids, consists of their ability to inhibit the cleavage of α-tubulin by trypsin [282].

Actin filaments are one of the major targets of cyanometabolites. In a similar manner to the microtubules, they exhibit a polymeric nature, being formed by monomers of globular actin (G-actin). The helical aggregation of these proteins occurs after their binding with ATP molecules, and it results in the formation of short chains of F-actin, which, with the aid of proteins, acquire stabilization [283]. Tolytoxin exhibits cytostatic effects against various cell types; among them are breast, human epidermoid, and ovarian carcinoma cells, as well as neural cells [74,273]. Its action mechanism involves the inhibition of actin filament formation and the depolymerization of preformed filaments, showing no effect on intermediary filamentous and microtubules. The core moiety of the macrolide directly interacts with the actin filaments; however, its tail affects the globular actin [284]. Swin, which displays a high degree of structural similarity to Tolytoxin, also exerts its toxicity by disrupting the actin cytoskeleton. The dimer is capable of binding to two G-actin molecules at the same time, thus leading to the formation of a tertiary complex. The sequestration of such proteins inhibits actin filament synthesis [248,285]. Conversely, Hectochlorin and Lyngbyabellin B promote actin polymerization [76].

### 4.3. Other Targets

The members of the Laxas family are known for showing significant differences in the action mechanism. Some are very cytotoxic, activating the apoptotic pathway through topoisomerase enzyme inhibition, whereas others, although not capable of blocking cell proliferation, can act by inducing autophagy, which can lead to the elimination of dysfunctional proteins or damaged organelles [124,286]. Laxaphycins B and B3 are among those with deleterious effects on the cell. Neuroblastoma cells exposed to these peptides have their Annexin A5 and Caspase 03 levels considerably enhanced. The expression of the former protein on the cell surface is related to the promotion of pro-apoptotic mechanisms, whereas the production of the second protein can lead to the activation of several apoptotic substrates that result in cell death [123]. L-Val^8^-laxaphycin A, D-Val^9^-laxaphycin A, [des-Gly] acyclolaxaphycin, acyclolaxaphycins B and B3, and [des-(Ala4-Hle5)] acyclolaxaphycins B and B3 have been described as inducers of autophagy. Mitochondria seem to be the primary target of these peptides since the initial cell response to these metabolites involves the reduction of ATP and reactive oxygen species production; this subsequently results in the activation of the AMP-activated protein kinase, whose main role is to maintain energy homeostasis. Once activated, the enzyme is likely to be responsible for ATP recovery after 24 h by inducing autophagic flux; this provides energy and substrates to the cell through catabolic processes [123,124].

Apart from topoisomerase, the fungicidal cyanometabolites can inhibit other enzyme groups, such as some proteases involved in pathogenesis, and it can inhibit the virulence of various fungi. Certain members of the *Candida* genus are known for releasing a battery of hydrolytic enzymes, which are responsible for digesting cell membranes, cells, and components of the immune system. Furthermore, they are capable of facilitating tissue adhesion and invasion [287]. Aspartyl proteinases are one of the most significant extracellular hydrolytic enzymes secreted by these microorganisms, and therefore, they have been the object of study for many researchers [287]. In accordance with the computational model generated by Manivannan and Muralitharan, the ABC transporter permease proteins of the cyanobacterium, *Microcoleus chthonoplastes* PCC 7420, effectively interact with the secreted aspartyl protease 5 of *C. albicans*, and thus, it can be utilized for the control of this pathogen [288]. Other cyanobacterial components have been reported as ligands of aspartyl proteases through docking analysis, such as the formic acid and nanonic acid identified in the ethanolic extract of the cyanobacteria, *M. aeruginosa* and *P. corium*, respectively [289]. Phenolic compounds extracted from *Spirulina* sp. LEB-18 dramatically diminished the amylase and protease content of the *F. graminearum*, and they were responsible for inhibiting strain growth [290].

The metabolite, Ambigol C, possesses an unknown mechanism in eukaryotic cells, but it is known that its addition to the cultivation media of *Serratia* sp. ATCC 39006 enhances prodiginine production through the increase in concentration of malonyl-CoA, and the uptake of L-proline, which act as precursors of this molecule [291]. Despite the low bioactivity of Ambigol C against fungi, some prodiginine analogs have been recognized for their antifungal and anti-algal activities [292,293]. Hence, the construction of microbial consortiums based on these properties could serve as a good strategy with regard to fungal growth control [291].

## 5. Regulation

### 5.1. Light

**Figure 28:**
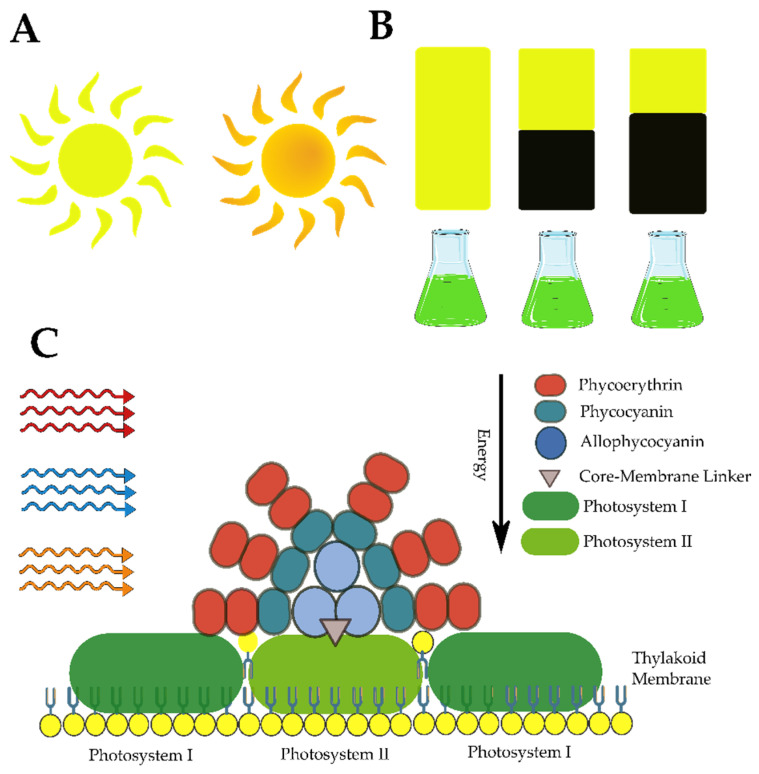
Light parameters normally investigated in studies concerning cyanobacterial growth and the production of secondary metabolites. (**A**) Light intensity, (**B**) Photoperiod, (**C**) Wavelength.

Cyanobacteria have established several strategies that permit their growth under distinct light conditions [294]. Currently, there are few studies that demonstrate the light intensity effect, nor the effect of light quality and photoperiods on the antifungal properties of such photosynthetic microorganisms. Each cyanobacterial strain can respond in a contrary or similar manner, with regard to alterations of these parameters (Figure 28) [295]. One example is light intensity, which, even though it is normally considered a limiting factor in the growth of various cyanobacteria, it has a reduced impact in some strains. Such differences are guaranteed by the morphological and physiological diversity that is present among members of this phylum [296,297].

The photoperiod (Figure 28B) has a strong influence on the behavior of cyanobacteria, affecting their growth and secondary metabolite biosynthesis [298]. As illustrated by Patterson and Bollis in 1993 [299], the influence of several abiotic factors on the production of Scytophycins by the cyanobacterium, *S. ocellatum* ATCC 55232, indicates that time in the light favors the biosynthesis of these peptides in such a way that the quantity of Tolytoxin that was produced was fivefold higher when the cyanobacterium was incubated in constant light; it was cultivated during an 8 h period of light. Cryptophycin content per unit of biomass also diminishes as the photoperiod decreases. The continuous use of light yields nearly 50 µg of the peptide per mg of dried biomass of the strain *Nostoc* sp. ATCC 53789, whereas only about 30 µg of the same material was obtained during 16 h of light [300]. Likewise, continuous light incubation (24-L) offers the best conditions for the production of the fungicide Majusculamide C, from *A. laxa*, followed by 16 h of light and 8 h of dark (L: D-8:16), 8 h of light, 16 h of dark (L: D-8:16), and 24 h of darkness (24-D) [301]. One plausible explanation for this correlation lies in the fact that the increase in the length of the light period affords a higher quantity of quantum available for photosynthesis; this results in a higher carbohydrate accumulation that can act as a carbon and energy source for the construction of fungicidal biomolecules [302]. These observations differ from those reported by Gupta and his research group [187] who detected a positive contribution during the dark period with regard to anti-fungal chitinase production from *A. fertilissima* RPAN1. Growth retardation and photosynthetic efficiency reduction were also observed in this strain when the light period was shortened, and the aforementioned effects are likely to be related to this event [187]. Similarly, Ibraheem and coworkers found that the L: D-16:8 cycle favors the release of a higher quantity of bioactive compounds against *C. albicans* IMRU 3669, *A. alternata*, *A. flavus* IMI 111023, *F. solani,* and *Pythium* sp. from the cyanobacterium, *C. minutus*, as compared with the conditions of 24-L and 24-D [303].

The light quality (Figure 28C) is another decisive factor in cyanobacterial physiology, as it is crucial for the equilibrium between photosystems II and I. Both apparatuses utilize an antenna formed by pigments to arrest light energy; however, they can be distinguished by the diversity and abundance of these dyes. Hence, some wavelengths are preferentially absorbed by the determined photosystem. Blue light, for example, is better captured by photosystem I inasmuch as this complex is composed of 96 chlorophyll molecules, whereas photosystem II only has 35. On the other hand, orange light is favorably absorbed by photosystem II since its antenna is mainly formed from phycobiliproteins. Regarding the red light, both chlorophyll and phycobilisome possess the ability to capture it [304]. A study of the model cyanobacterium, *Synechocystis* sp. PCC 6803, has shown a negative impact on cell growth under blue light due to an imbalance between the two photosystems, which is caused by an energy surplus at PSI and a deficiency in the electron transfer chain [305]. The orange-red light is clearly preferred for obtaining the best growth rate for *Nostoc* sp. ATCC 53789, as well as the production of Cry, compared with the blue light, which offered the worst conditions for the production of the peptide [300]. Incubation with blue and green light does not coincide with the optimal growth of *S. ocellatum* ATCC 55232, but it has no significant effect on Scytos production [299].

The light intensity (Figure 28A) is the most investigated light parameter with regard to the expression of antifungal metabolites by cyanobacteria. Elevated light intensity has been correlated with the deceleration of growth in several cyanobacteria, and it is responsible for provoking cell death in certain situations following oxidative stress. From medium to very high light intensity (80–200 µmol photon m^−2^ s^−1^), *Nostoc* sp. ATCC 53789 exhibited considerably reduced growth, and the biosynthesis of Cry was also reduced [300]. Norharmane production in *C. minutus* is also determined by light intensity. At both 15 °C and 35 °C, incubation with 43.61 µmol photon m^−2^ s^−1^ ensures a better expression of the alkaloid in comparison with 98.9 µmol photon m^−2^ s^−1^ [306]. Similar light intensities were found to be responsible for increasing the antimicrobial potential of another strain of *C. minutus* [303].

### 5.2. Nutrients

Some bioproducts of cyanobacteria are dependent on the limitations of one or more nutrients [307,308,309,310,311]. For instance, the Scyto accumulation in *S. ocellatum* ATCC 55232 noticeably varies depending on the growing medium. The BG-11 and Allen media offer the cyanobacteria more favorable conditions for the production of macrolides, whereas cultivation with Gerloff, A3M7, C, and Volvox media results in a low yield [299]. With this in mind, Patterson and Bolis, in a subsequent investigation, evaluated the effects of the source and concentration of the BG-11 medium elements on Scyto formation using this strain [312]. Of the potassium phosphate quantity examined, concentrations above 1.7 mM resulted in a lower dry biomass yield and higher 6-Hydroxy-7-O-methylscytophycin E and 19-O-demethylscytophycin C production without causing any alterations to Tolytoxin accumulation [312]. Regarding the sulfur source, sulfite, sulfate, or thiosulfate were incorporated into the medium at equimolar concentrations, which led to a similar amount biomass and Scyto being produced [312]. However, the complete deprivation of this element culminated in the reduced production of macrolides, and it did not significantly alter the growth of the cyanobacterium [312]. On the other hand, both the nitrogen source and concentration had an impact on biomass and Scyto accumulation. Of the nitrogen sources evaluated, nitrate provided the greatest biomass and Scyto yields, whereas the use of urea, amino acids, and indole occasionally reduced growth and Scyto formation [312]. These data match with those reported for another *Scytonema* strain’s (TISTR 8208) growth on polyurethane foam, wherein nitrate was the best nitrogen source, and its concentration was positively correlated with antibiotic production [308]. The cultivation of TISTR 8208 in the BG11 medium resulted in the improved production of the antagonistic compound in comparison to the Allen and BGA media. However, the supplementation of the latter medium with nitrogen elicited greater antimicrobial production, even exceeding the yield obtained in the BG11 medium [308]. The enrichment of the same medium, also enriched with 1.5 g L^−1^ of nitrate and phosphate, was responsible for boosting the antagonistic activity of the nitrogen-fixing cyanobacterium, *Calothrix* sp. TISTR 8906, against the phytopathogen, *Macrophomina phaseolina* [313]. The optimization of such activity allowed the efficient use of the cyanobacterial extract in the *M. phaseolina* control, which targeted mung beans at a concentration that was similar to that reported for the commercial fungicide, mancozeb [313].

The BG-11 medium was also the preferred medium for producing one inhibitory compound against *A. niger* and *A. flavous* using *N. muscorum*, as compared with Benecke’s solution and Rhode and Chu media [257]. The maximum production of this metabolite was acquired after doubling the nitrate concentration and reducing the quantity of the remaining constituents by half. Conversely, in the opposite situation, wherein the nitrate quantity was diminished by half and the other elements increased by 100%, the antimicrobial activity reached its minimum production value [257]. A similar finding was observed for *Symphyonema bifilamentata* sp. nov. 97.28. Its initial cultivation in a Z-medium led to the detection of Ambis A–B, whereas the utilization of the BG11 medium allowed for Ambis A, C, D, and E identification [109,221,258]. In the same sense, the full replacement of chlorin, either via bromine or iodine atoms through the alteration of the BG-11 medium’s composition, resulted in the total loss of Ambigols. These experimental data suggest that the halogenase enzyme involved in the production of such phenolic compounds is highly specific to chlorine [109]. The influence of halide ions has also been the object of study for Carbamidocyclophanes biosynthesis. Different from Ambigols, the halogenase enzyme involved in the Carbamis’ formation seems to have low substrate specificity; this is due to the cultivation of the cyanobacterium, *Nostoc* sp. CAVN2, in a KBr or KCL-enriched medium, which resulted in the identification of brominated and chlorinated Carbamis, respectively. The average total content of the two types of halogenated Carbamis was nearly the same. However, the cultivation of the strain in a medium supplemented with both halogens at equimolar concentrations demonstrated the microorganism’s particular preference for chloride, whereas the absence of these halogens resulted in a 50-fold reduction in the Carbamis’ total content [242].

The cyanobacterial siderophores are also very sensitive to nutritional alterations that occur in the environment. These chelators are known for their low molecular weight and high affinity with iron; thus, they play an essential role in the survival of cyanobacteria, which, due to their photosynthetic nature, require such elements in much larger quantities than those documented for a non-photosynthetic organism. Some cyanobacteria use siderophores as antibiotics [314]. The thermotolerant cyanobacterium, *Phormidium* sp., investigated by Fish and Codd, produces siderophore-like metabolites with antifungal properties against *C. albicans*. Although temperature, nitrate, and nitrilotriacetic acid concentrations had been identified as one of the main regulators involved in the production of these compounds, the iron (III) chloride concentration exerted the greatest effect [309].

In addition to the abiotic factors mentioned above, Tolytoxin production can be up-regulated in a dose-dependent manner with the addition of fungal extracts from *P. notatum* and *C. spathiphyllum* in the medium. Individual analyses of the extract components of the Tolytoxin accumulation allowed the identification of chitin, carboxymethylcellulose, and pectin as the components responsible for the elicitor activity. The presence of acetyl or carboxymethyl functional groups seems to be correlated with this property since the chitosan and cellulose, which are, respectively, deacetylated and dealkylated forms of chitin and carboxymethylcellulose, display weak or no elicitor potential. None of the evaluated monosaccharides, such as glucose and mannose, were capable of altering the expression of the macrolide. This study was important so that a comparison of Tolytoxin’s ecological role, with the reported ecological role of phytoalexin, whose production occurs in response to fungal attacks, can be conducted [315]. In opposition to the previous finding, Nowruzi and coworkers demonstrated that glucose supplementation, combined with high light intensity, has a positive impact on the antimicrobial properties of the FSN_E and ASN_M *Nostoc* strains against *A. niger* ATCC 16404 and *C. albicans* ATCC 10231. While growth under autotrophic or mixotrophic conditions using sucrose does not favor the production of antimicrobials [316].

### 5.3. pH

Little is known about how pH disturbs the antifungal properties of cyanobacteria. In certain situations where there is a change in the composition of the medium, this parameter has been ignored. Therefore, it is sometimes unclear as to whether the change in cyanobacterial bioactivity is a direct response to the supplementation or replacement of the given element, or a combined effect that occurs with pH changes. An increase in Norharmane production due to the cyanobacterium, *C. minutus*, under neutral pH conditions, was observed by Karan and coworkers. At pH values of 5 and 9, the synthesis of the metabolite undergoes a reduction of about 98.35 and 63.8%, respectively [306]. Chaudhary, Prasanna, and Bhatnagar (2012) [317] demonstrated that the growth of *Anabaena* strains differ under distinct pH conditions, thus resulting in significant changes in the fungicidal potential of the cell-free filtrate obtained from these cyanobacteria. Using *P. debaryanum* and *F. oxysporum* during the experiment, it was noted that such consequences vary depending on the target microorganism utilized. For example, although a pH of 5.5 provides the best conditions for the acquisition of antimicrobial metabolites from the RP69 strain that works against *P. debaryanum*, the same pH has no elicitor effect on the antagonistic activity that works against *F. oxysporum*, compared with the other tested pH values (7.5 and 9.5) [317]. On some occasions, the optimum pH for cyanobacterial growth offered the most favorable environment for the synthesis of the biocide compounds, *S. maxima* and *S. ocellatum*; indeed, their best growth and inhibitory compound production occurred between the pH values of 8.0–8.5 [299,318].

### 5.4. Temperature

Various studies have documented the modulating effect of temperature on cyanobacterial behavior [319,320,321,322]. Low temperatures are responsible for reducing photosynthetic electron transportation, whereas high temperatures significantly modify carbon dioxide and nitrogen fixations [323]. The effects of such events, in addition to deeply affecting the primary metabolism, can alter the secondary metabolism, which is involved in toxin biosynthesis [324,325]. Although some compounds belong to the same family, their production may be differently affected when the cyanobacterium is exposed to temperature variations, such as the Carbamidocyclophanes obtained from *Nostoc* sp. CAVN10. The maximum accumulation of the highly cytotoxic variants A–C and E is observed between 24 to 28 °C, whereas the production of the low-active variant D is not significantly affected by temperatures ranging from 24 to 33 °C. On the other hand, the optimum temperature for strain growth is considerably higher (33 °C) [240]. The contrast between the best temperature for growth and fungicide production has been reported in another study wherein the exopolysaccharide quantity and anti-*Candida* activity, extracted from the thermophilic cyanobacterium *Gloeocapsa* sp., decreased progressively with temperature increases and the increased growth rate [326]. The generation of Tolytoxin using *S. ocellatum* FF-66-3 is also not directly proportional to the growth and temperature parameters. Although the strain archived its maximum growth at 20 °C, the metabolite achieved a better accumulation at 25 °C with 6-Hydroxy-7-O-methylscytophycin E. The aforementioned bacteria, as well as 19-O-demethylscytophycin C, have their production drastically diminished above 35 °C [299]. In different circumstances, the rise in temperature simultaneously favors Norharmane production and the growth of *C. minutus* [327].

## 6. Final Considerations

The fungicidal compounds extracted from cyanobacteria are very structurally diverse, and they have been detected in distinct ecosystems. They are mostly peptides, and their main source is filamentous cyanobacteria. Some of these substances are exclusively produced by a certain group of cyanobacteria, such as the cryptophycins, which have only been encountered in cyanobacteria of the genus, *Nostoc*. Lifestyle also seems to be a determining factor in the production of certain compounds; for example, the hapalindole-type alkaloids, which are mainly found in stigonematalean cyanobacteria that are isolated from freshwater and terrestrial environments.

The production of these fungicides is affected by environmental factors. The study of such parameters has not only aided the optimization of the production of these compounds at a level that allows for their preclinical and clinical investigation, but it has also made their ecological role clearer. Among the investigated parameters are the concentration of certain nutrients, the light quality, quantity, temperature, and pH. The rich enzymatic apparatus, as well as the low-substrate tolerance of some enzymes, have justified the existence of several variants from the same group in just a single strain. The biosynthesis of a diversity of congeners has been proposed as an adaptive strategy against parasites with a rapid evolution.

The mechanism of action employed by such antifungal agents can be very diverse, even among members belonging to the same family. The majority of studies have utilized mammalian cells to elucidate the effect of these metabolites since they have also been evaluated for their anticancer properties. Cell membrane and cytoskeleton components have been one of the main targets investigated. Despite some of these agents sharing the same target, they can exhibit distinct pathways to kill the cells. The majority of antifungal metabolites of cyanobacterial origin do not have their action mechanism elucidated, and thus, more studies are required.

## Figures and Tables

**Table 1:** List of antifungal peptides reported in cyanobacteria.

Compound	Molecular Weight	Cyanobacterial Strain (Family)	Source	Location	Ref
Schizotrin A	1490.71	*Schizothrix* sp. IL-89-2 (Schizotrichaceae)	Terrestrial	Kfar Azar (Israel)	[33]
Pahayokolides A	1472.68	*Lyngbya* sp. 15-2 (Oscillatoriaceae)	Freshwater	Everglades (Florida, U.S)	[38]
Muscotoxins A	1211.41	*Desmonostoc muscorum* CCALA 125 (Nostocaceae)	Terrestrial	Dlouhá ves (Chelcice, Czech Republic)	[39,40]
Muscotoxins B	1225.45				
Calophycin	1249.48	*Calothrix fusca* EU-10-1 (Rivulariaceae)	Freshwater	Island of Oahu (Hawaii, U.S)	[36]
Puwainaphycin F	1146.34	*Cylindrospermum alatosporum* CCALA 988 (Aphanizomenonaceae)	Terrestrial	Riding Mountain National Park (Manitoba, Canada)	[41]
Minutissamide A	1118.28	Nostocales LEGE 17548	Freshwater	Lagoa de Mira (Mira, Portugal)	[42]
		*Anabaena minutissima* UTEX 1613 (Aphanizomenonaceae)	Terrestrial	South Texas, USA	[43,44]
Nostofungicidine	885.57	*Nostoc commune* (Nostocaceae)	Terrestrial	Gulf of Finland (Porkkala, Finland)	[45]
Anabaenolysin A	558.63	Benthic *Anabaena* strains (Aphanizomenonaceae)	Marine	Island of Oahu (Hawaii, U.S)	[46,47]
Anabaenolysin B	560.64				
Laxaphycin A	1196.48	*Anabaena laxa* FK-1-2 (Aphanizomenonaceae)	Terrestrial	Island of Oahu (Hawaii, U.S)	[48]
		*Hormothamnion* *Enteromorphoides* (Nostocaceae)	Marine	Key West (Florida, U.S)	[49]
		*Lyngbya majuscula* (Oscillatoriaceae)	Marine	Moorea atoll (French Polynesia)	[50]
		*Anabaena torulosa* (Aphanizomenonaceae)	Marine	Moorea atoll (French Polynesia)	[51]
Laxaphycin B	1395.70	*A. laxa* FK-1-2 (Aphanizomenonaceae)	Terrestrial	Island of Oahu (Hawaii, U.S)	[48]
		*L. majuscula* (Oscillatoriaceae)	Marine	Moorea atoll (French Polynesia)	[49]
		*A. torulosa* (Aphanizomenonaceae)	Marine	Moorea atoll (French Polynesia)	[50]
Laxaphycin C	1379.69	*A. laxa* FK-1-2 (Aphanizomenonaceae)	Terrestrial	Island of Oahu (Hawaii, U.S)	[48]
Scytocyclamide A	1223.52	*Scytonema hofmannii* PCC 7110 (Scytonemataceae)	Terrestrial	Bermuda (United Kingdom)	[52]
Scytocyclamide A2	1208.51				
Scytocyclamide B	1367.65				
Scytocyclamide B2	1352.64				
Scytocyclamide B3	1336.64				
Scytocyclamide C	1351.65				
Heinamides	1186.65–1518.85	*Nostoc* sp. UHCC 0702 (Nostocaceae)	Freshwater	Villähteen Kukkanen (Nastola, Finland)	[53]
Hormothamnin A	1196.48	*H. Enteromorphoides* (Nostocaceae)	Marine	La Parguera (Puerto Rico Caribbean)	[54]
Microcolin A	747.96	*Lyngbya* cf. polychroa (Oscillatoriaceae)	Marine	Coast of Hollywood (Florida, U.S)	[55]
Microcolin B	731.96	*L. majuscula* (Oscillatoriaceae)	Marine	La Blanquilla Island (Venezuela)	[56]
		*Moorea producens* (Oscillatoriaceae)	Marine	Playa Kalki (Curaçao)	[57]
		*Oscillatoria* sp. LP16 (Oscillatoriaceae)	Marine	Broward County (Florida, U.S)	[58]
Lobocyclamide A	1198.45	*Lyngbya confervoides* (Oscillatoriaceae)	Marine	Cay Lobos (Bahamas, Caribbean)	[59,60]
Lobocyclamide B	1398.70				
Lobocyclamide C	1370.65				
Nostocyclamide	474.55	*Nostoc* sp. 31 (Nostocaceae)	Freshwater	New Jersey (United States	[61,62]
Hectochlorin	665.59	*L. majuscula* UOG VP417 (Oscillatoriaceae)	Marine	Apra Harbor (Guam)	[63]
		*M. producens* JHB (Oscillatoriaceae)		Hector Bay (Jamaica)	[64]
Lyngbyabellin B	665.59	*L. majuscula* JHB-22 (Oscillatoriaceae)	Marine	Hector Bay (Portland, Jamaica)	[65]
		*M. producens* RS05 (Oscillatoriaceae)		Sharm el-Sheikh (Egypt)	[66]
Hassallidin A	1382.52	*Hassallia* sp. B02-07 (Tolypothrichaceae)	Marine	Orrido Clough (Bellano, Italy)	[67,68]
Hassallidin B	1528.66				
Hassallidin D	1865.00	*Anabaena* sp. SYKE748A (Aphanizomenonaceae)	Marine	Tuusulanjarvi Lake (Tuusulanjarvi, Finland)	[69]
Hassallidin E	1410.58	*Planktothrix serta* PCC 8927 (Microcoleaceae)	Marine	Berre le Clos (França)	[70]
Balticidin A	1787.28	*Anabaena cylindrica* Bio33 (Aphanizomenonaceae)	Marine	Baltic Sea (Rügen Island, Germany)	[71]
Balticidin B	1769.26				
Balticidin C	1752.83				
Balticidin D	1734.81				
Tolybyssidin A	1465.80	*Tolypothrix byssoidea* EAWAG 195 (Tolypothrichaceae)	Marine	Pokhara (Nepal)	[72]
Tolybyssidin B	1491.86				
Cryptophycin A	655.19	*Nostoc* sp. ATCC 53789 (Nostocaceae)	Terrestrial	Isle of Arran (Scotland)	[73,74]

**Table 2:** Comparison of activity between cyanobacterial antifungals and commercial drugs. The activity is presented in minimum inhibitory concentration (µM/mM/µg mL^−1^/mg mL^−1^), Half-maximal inhibitory concentration (IC_50_), diameter of the inhibition halo (mm), or percentage inhibition of radial growth (%).

Metabolite	Target	Activity		Ref
		Metabolite	Control	
Anabaenolysin A	*C. albicans* HAMBI 261	-	-	[47]
Anabaenolysin B	*C. albicans* HAMBI 261	-	-	
Balticidin A	*Candida maltosa* SBUG700	12 mm	-	[71]
Balticidin B		15 mm	-	
Balticidin C		9 mm	-	
Balticidin D		18 mm	-	
Calophycin 1.2 µg	*Aspergillus oryzae*	13 mm	No activity Amphotericin B	[36]
	*Penicillium notatum*	12 mm	No activity Amphotericin B	
	*S. cerevisiae*	12 mm	9 mm Amphotericin B	
Calophycin	*Trichophyton mentagrophytes*	2 µM	-	
	*Aspergillus fumigatus*	1 µM	1.35 µM Amphotericin B	
	*C. albicans*	1µM	0.67 µM Amphotericin B	
Cryptophycin A	*Rhizonucor miehei*, *Scopulariopsis communes*, *Trichoderma lignorum*, *Verticilium serrae*, *Cryptococcus albidus*, *Cryptococcus laurentii*, *Ustilago zea*, and *Cercospora beticola*.	10–15 mm	-	[74]
	*A. fumigatus, Cephalosporium* sp., *F. oxysporum, and Ceratocystis ulmi*	16–20 mm	-	
	*Alternaria solani, Aspergillus flavus, Aspergillus niger, Botrytis ali, Penicillium* sp., *Cochliobolus miyabeanus, Phona* sp.	20–25 mm	-	
	*Cryptococcus neoformans* MY1051 and MY1146	≤0.1 µM	<1.1 µM Amphotericin B	
	*C. neoformans* strains	0.046 µM		
Hassallidin A	*C. albicans* strains *Candida guillermondii* ATCC 90877 *C. tropicalis* ATCC 750	2.9 µM	0.23–0.91 µM Caspofungin	[67,68,75]
	*Cryptococcus neoformans* *Trichosporon asahii* *Trichosporon mucoides*		7.3 -29.3 µM Caspofungin	
	*Candida glabrata* strains *Candida parapsilosis* strains *C. tropicalis* strains *Candida krusei* strains	5.2 µM	0.23–0.91 µM Caspofungin	
	*Aspergillus niger* *Ustilago maydis* *Penicillium* sp. *Fusarium sambucium*.	-	-	
Hassallidin B	*C. parapsilosis* ATCC 22019 *C. krusei* ATCC 6258 *C. albicans* strains *C. tropicalis* ATCC 90874	5.2 µM	0.23–0.91 µM Caspofungin	
	*C. neoformans* strains		7.3–29.3 µM Caspofungin	
	*C. albicans* ATCC 24433 *C. glabrata* strains *C. parapsilosis* ATCC 90018 *C. tropicalis* ATCC 750 *C. krusei* ATCC 90878	10.5 µM	0.23–0.91 µM Caspofungin	
	*Aspergillus niger* *Ustilago maydis* *Penicillium* sp. *Fusarium sambucium*.	-	-	
	*A. fumigatus* and *C. albicans*	3.1 µM	-	
Hassallidin D	*C. albicans* ATCC 11006 *C. albicans* ATCC 10231 *C. krusei* ATCC 6258	1.5 µM	-	[69]
Hassallidin D 10 μg	*Cryptococcus albidus* ATCC 10666	17 mm	-	[69]
	*Filobasidiella neoformans* ATCC 10226	11 mm	-	
Hassallidin D Linear Form	*C. albicans* ATCC 11006	20 µM	-	
Hassallidin E	*C. albicans* CBS562	22.7 µM	-	[70]
	*C. neoformans* H99		-	
	*C. parapsilosis* ATCC22019		-	
	*C. krusei* ATCC6258		-	
Hectochlorin 100 µg	*C. albicans*	16 mm	-	[76]
Heinamides	*Aspergillus flavus* FBCC 2467	-	-	[53]
Hormothamnins (10 µg)	*C. albicans*	7–11 mm	-	[77]
Laxaphycins A + B (25 + 25 µg)	*A. oryzae*	29 mm	-	[48]
	*C. albicans*	23 mm	-	
	*P. notatum*	30 mm	-	
	*S. cerevisiae*	22 mm	-	
	*T. mentagrophytes*	30 mm	-	
Laxaphycin B (50 µg)	*A. oryzae*	19 mm 45.8 µM 4.6 µM (8.1 µM of Laxa A)		
	*C. albicans*	8 mm	-	
	*S. cerevisiae*	12 mm	-	
	*T. mentagrophytes*	9 mm	-	
Laxaphycin C (50 µg)	*A. oryzae*	14 mm	-	
	*C. albicans*	9 mm	-	
	*P. notatum*	11 mm	-	
	*S. cerevisiae*	12 mm	-	
Lobocyclamide A (150 µg)	*C. albicans* 96-489	7 mm	-	[59]
Lobocyclamide B (150 µg)	*C. albicans* 96-489	8 mm	-	
	*C. glabrata*	6 mm	-	
Lobocyclamide C (150 µg)	*C. albicans* 96-489	10 mm	-	
	*C. glabrata*	8 mm	-	
Lyngbyabellin B (100 µg)	*C. albicans* ATCC 14053	10.5 mm	-	[78]
Microcolin A	*Dendryphiella salina* SIO *D. salina* EBGJ	>250 µM	3.4 µM Amphotericin B	[55]
Microcolin B				
Minutissamide A	*A. fumigatus*	37.5 µM	-	[43]
	*Alternaria alternata*	75 µM	-	
Muscotoxins A	*Candida friedrichii*	61.9 µM	26.1 µM Fluconazole	[40]
	*Trichoderma harzianum*	31.5 µM	13.1 µM Fluconazole	
	*Bipolaris sorokiniana*	NA	NA Fluconazole	
	*Alternaria alternata*	0.5 µM	6.5 µM Fluconazole	
	*Monographella cucumerina*	1.9 µM	6.5 µM Fluconazole	
	*A. fumigatus*	1.9 µM	104.5 µM Fluconazole	
	*Chaetomium globosum*	15.5 µM	6.5 µM Fluconazole	
	*Fusarium oxysporum*	61.9 µM	26.1 µM Fluconazole	
	*S. sclerotiorum*	41.3 µg *	-	
Muscotoxins B	*S. sclerotiorum*	20.4 µg *	-	
Nostocyclamide	*S. cerevisiae*	-	-	[62]
Nostofungicidine	*Aspergillus candidus*	1.8 µM	-	[45]
Pahayokolides A	*S. cerevisiae*	20 mm	-	[38]
Puwainaphycin F	*C. albicans* HAMBI 261	5.5 µM	-	[79]
	*S. cerevisiae* HAMBI 1164		-	
Schizotrin A (13.4–16.7 nM)	*S. cerevisiae* *C. albicans*	7 mm	-	[33]
	*C. tropicalis*	9 mm	-	
	*R. rubra*	8 mm	-	
	*S. rolfsii* *R. solani*	25–28%	-	
	*C. gloeosporioides*	47%	-	
Schizotrin A 33.5 nM	*F. oxysporum*	37%	-	
Scytocyclamide A 200 µg	*A. flavus* FBCC 2467	10 mm	-	[52]
Scytocyclamide A2 200 µg		7 mm	-	
Scytocyclamide B 600 µg		23 mm	-	
Scytocyclamide B2 85 µg		10 mm	-	
Scytocyclamide B3 85 µg		20 mm	-	
Scytocyclamide C 160 µg		22 mm	-	
Scytocyclamide A + B 100 + 300 µg		36 mm	-	
Scytocyclamide A + C 100 + 80 µg		33 mm	-	
Scytocyclamide A2 + B2 100 + 43 µg		24 mm	-	
Scytocyclamide A2 + B3 100 + 43 µg		25 mm	-	
Scytocyclamide B + C 300 + 80 µg		23 mm	-	
Tolybyssidin A	*Candida albicans*	21.8 µM	19.2 µM Miconazole	[72]
Tolybyssidin B		42.9 µM		
7-Deoxysedoheptulose	*S. cerevisiae*	50 µM	590 μM Glyphosate	[80]
Polysaccharide 10 µg	*T. rhizoctonia*	15 mm	23 mm Amphotericin	[81]
	*F. solani*	16 mm	27 mm Amphotericin	
	*F. oxysporum*	15 mm	28 mm Amphotericin	
	*A. niger*	17 mm	25 mm Amphotericin	
	*C. albicans* ATCC 90028	15 mm	20 mm Amphotericin	
Polysaccharide	*Botrytis cinerea*	1064 mg mL^−1^ (EC_50_)	-	[82]
Polysaccharide	*A. niger*	707 mg mL^−1^	-	[83]
Chlorosphaerolactylate A	*C. parapsilosis SMI416*	3 mM		[84]
Chlorosphaerolactylates B–C		3.3 mM	-	
Chlorosphaerolactylate D		5.5 mM	-	
Majusculoic Acid	*C. albicans* ATCC 14503	8 µM	1 µM Fluconazole	[85]
	*C. glabrata*	19.3 µM	-	
Mirabilenes A–F 10 μg	*P. notatum*	10–15 mm	-	[86]
	*A. oryzae*			
Lyngbic Acid	*Fusarium* sp.	2.1 µM^IC^_50_	-	[87]
	*Lindra thalassiae*	2.7 µM^IC^_50_	-	
	*D. salina*	2.9 µM^IC^_50_	-	
Tanikolide 100 μg	*C. albicans*	13 mm	-	[88]
Ambiguine A	*C. albicans*	6.1 µM	0.34 µM Amphotericin B	[89,90,91]
	*T. mentagrophytes*	24.6 µM	0.06 µM Tolnaftate	
	*A. fumigatus*	196.6 µM	2.3 µM Amphotericin B	
Ambiguine B	*C. albicans*	5.9 µM	0.34 µM Amphotericin B	
	*T. mentagrophytes*	5.9 µM	0.06 µM Tolnaftate	
	*A. fumigatus*	47.3 µM	2.3 µM Amphotericin B	
Ambiguine C	*C. albicans*	3.2 µM	0.34 µM Amphotericin B	
	*T. mentagrophytes*	1.6 µM	0.06 µM Tolnaftate	
	*A. fumigatus*	>20.6 µM	2.3 µM Amphotericin B	
Ambiguine D	*C. albicans*	2.8 µM	0.34 µM Amphotericin B	
	*T. mentagrophytes*	1.4 µM	0.06 µM Tolnaftate	
	*A. fumigatus*	176.6 µM	2.3 µM Amphotericin B	
Ambiguine E	*C. albicans*	5.7 µM	0.34 µM Amphotericin B	
	*T. mentagrophytes*	5.7 µM	0.06 µM Tolnaftate	
	*A. fumigatus*	>183.1 µM	2.3 µM Amphotericin B	
Ambiguine F	*C. albicans*	2.7 µM	0.34 µM Amphotericin B	
	*T. mentagrophytes*	2.7 µM	0.06 µM Tolnaftate	
	*A. fumigatus*	>175.8 µM	2.3 µM Amphotericin B	
Ambiguine G	*C. albicans*	>100 µM	0.03 µM Amphotericin B	
Ambiguine I	*S. cerevisiae*	1.5 µM	0.57 µM Puromycin	
	*C. albicans* ATCC 90028	1.5 µM	1.7 µM Amphotericin B	
Ambiguine K	*C. albicans*	<0.9 µM	0.03 µM Ketoconazole	[92]
Ambiguine L		<1.0 µM		
Ambiguine M		1.1 µM		
Ambiguine N		<1.0 µM		
Ambiguine O		<1.0 µM		
Ambiguine P		32.9 µM		
Anhydrohapaloxidole A	*C. albicans*	1.9 µM	0.12 µM Amphotericin B	[93]
Carriebowlinol	*Fusarium sp.*	0.2 µM^IC^_50_	-	[87]
	*L. thalassiae*	0.4 µM ^IC^_50_	-	
	*D. salina*	0.5 µM ^IC^_50_	-	
Fischerindole L	*C. albicans*	1.2 µM	0.12 µM Amphotericin B	[93]
12-epi-fischerindole U	*C. albicans* SC5314	1.2 µM	-	[94]
12-epi-fischerindole G		1.6 µM	-	
13R-Bromo 12-epi-fischerindole U		2.5 µM	-	
Fischambiguine A	*C. albicans*	15.3 µM	0.12 µM Amphotericin B	[92]
Hapalindole A	*C. albicans*	3.7 µM	-	[95]
	*T. mentagrophytes*	3.7 µM	-	
Hapalindole B	*C. albicans*	>53.9 µM	-	
	*T. mentagrophytes*	>53.9 µM	-	
Hapalindole C	*C. albicans*	2.1 µM	-	
	*T. mentagrophytes*	2.1 µM	-	
Hapalindole D	*C. albicans*	59.4 µM	-	
	*T. mentagrophytes*	59.4 µM	-	
Hapalindole E	*C. albicans*	0.9 µM		
	*T. mentagrophytes*	1.8 µM	-	
Hapalindole F	*C. albicans*	53.9 µM	-	
	*T. mentagrophytes*	53.9 µM	-	
Hapalindole G	*C. albicans*	29.5 µM	-	
	*T. mentagrophytes*	7.4 µM	-	
Hapalindole H	* C. albicans *	32.9 µM	-	
	* T. mentagrophytes *	4.1 µM	-	
	* C. albicans *	<0.6 µM	-	
Hapalindole A	*C. albicans*	1.2 µM	0.12 µM Amphotericin B	[93]
Hapalindole J		0.7 µM	0.12 µM Amphotericin B	
Hapalindole X		2.5 µM	0.12 µM Amphotericin B	
Hapalonamide H		<0.6 µM	0.12 µM Amphotericin B	
Nostocarboline	*S. cerevisiae* A-136	4.6 µM	7.9 µM Chlorhexidine	[96]
	*C. albicans* T-3419	2.3 µM		
Norharmane	*C. albicans* ATCC 10231	237 µM	-	[97]
Tjipanazole A	*C. albicans*	-	-	[98]
	*T. mentagrophytes*	-	-	
	*A. flavus*	-	-	
Welwitindolinone A isonitrile	*A. oryzae*	-	-	[99]
	*P. notatum*	-	-	
	*S. cerevisiae*	-	-	
Fischerellin A 611.9 µM	*U. appendiculatus*	100%	-	[100]
Fischerellin A 2.44 µM	*Erysiphe graminis*	100%	-	
	*Phytophthora infestans*	80%	-	
	*Pyricularia oryzae*	80%	-	
	*Monilinia fructigena*	80%	-	
	*Pseudocercosporella herpotrichoides*	30%	-	
Kalkipyrone A	*S. cerevisiae* ABC16-Monster	14.6 µM^IC^_50_	-	[101]
Kalkipyrone B		13.4 µM^IC^_50_	-	
Yoshinone A		63.8 µM^IC^_50_	-	
Carbamidocyclophane A	*C. albicans*	5.5 µM	-	[102]
Carbamidocyclophane B		1.3 µM	-	
Carbamidocyclophane F		2.9 µM	-	
Scytophycin A	*Saccharomyces pastorianus*	17 mm	-	[103]
	*Neurospora crassa*	25 mm	-	
	*Candida albicans*	19 mm	-	
	*Pythium ultimum*	>30 mm	-	
	*R. solani*	23 mm	-	
	*Sclerotina homoeocarpa*	30 mm	-	
Scytophycin B	*S. pastorianus*	20 mm	-	
	*N. crassa*	27 mm	-	
	*C. albicans*	22 mm	-	
	*P. ultimum*	30 mm	-	
	*R. solani*	>30 mm	-	
	*S. homoeocarpa*	>30 mm	-	
Scytophycin C	*S. pastorianus*	17 mm	-	
	*N. crassa*	30 mm	-	
	*C. albicans*	22 mm	-	
	*P. ultimum*	12 mm	-	
	*R. solani*	30 mm	-	
	*S. homoeocarpa*	32 mm	-	
Scytophycin D	*S. pastorianus*	12 mm	-	
	*N. crassa*	25 mm	-	
	*C. albicans*	19 mm	-	
	*P. ultimum*	27 mm	-	
	*R. solani*	30 mm	-	
	*S. homoeocarpa*	26 mm	-	
Scytophycin E	*S. pastorianus*	23 mm	-	
	*N. crassa*	36 mm	-	
	*C. albicans*	21 mm	-	
	*P. ultimum*	40 mm	-	
	*R. solani*	46 mm	-	
	*S. homoeocarpa*	35 mm	-	
Tolytoxin	*C. albicans* A26 *T. mentagrophytes* A23	8 nM	0.12–1 nM Nystatin	[104]
	*S. cerevisiae* *Phytophtora nicotianae* H729 *A. alternata* 1715 *Colletotrichum eoecodes* 1809	4 nM		
	*Bipolaris incurvata* 2118 *Caloneetria critalarae* 1809	2 nM		
	*Sclerotium rolfsii* 2133 *Thielaviopsis paradoxa* 1215	1 nM		
	*A. oryzae* *Phyllosticta capitalensis*	0.5 nM		
	*P. notatum* *R. solani* 1165	0.25 nM		
Swinholide A	-	-	-	[105]
Amantelide A 7.9 µM	*D. salina*	0%	100% Amphotericin B (67.6 µM)	[106]
	*L. thalassiae*	40%	21% Amphotericin B (67.6 µM)	
	*Fusarium* sp.	35%	6% Amphotericin B (67.6 µM)	
Sacrolide A	*Penicillium chrysogenum*	3.2 µM	-	[107,108]
	*S. cerevisiae*	25.9 µM	-	
	*C. albicans*	25.9 µM	-	
9-epi-sacrolide A	*S. cerevisiae*	≦ 25.9 µM	-	
	*P. chrysogenum*	≦ 25.9 µM	-	
15,16-dihydrosacrolide A	*S. cerevisiae*	25.8 µM	-	
	*P. chrysogenum*	25.8 µM	-	
Ambigol A 50 µg	*Microbotryum violaceum*	7 mm	20 mm Miconazole	[109]
	*Eurotium repens*	7 mm		
	*F. oxysporum*	8 mm		
	*Mycotypha microspora*	4 mm		
Ambigol C 50 µg	*M. violaceum*	5 mm		
4,4′-dihydroxybiphenyl	*C. albicans* ATCC 10231	171.8 µM	-	[97]
Parsiguine	*C. krusei* ATCC 44507	20 µg mL^−1^	-	[110]
Scytoscalarol	*C. albicans*	4 µM	-	[111]

*, the lowest dose to manifest its activity (µg).

**Table 3:** List of carbohydrates and their derivatives found in cyanobacteria.

Compound	Molecular Weight	Cyanobacterial Strain (Family)	Source	Location	Ref
Polysaccharide	-	*Nostoc Commune* (Nostocaceae)	Terrestrial	Nanbu County (SiChuan, China)	[83]
	-	*Phormidium versicolor* NCC 466 (Oscillatoriaceae)	Terrestrial	Sfax (Tunisia)	[81]
	-	*Anabaena* sp. BEA 0300B (Aphanizomenonaceae)	Terrestrial	Ajuy (Gran Canaria, Spain)	[82]
7-Deoxysedoheptulose	194.18	*Synechococcus elongatus* PCC 7942 (Synechococcaceae)	Freshwater	San Francisco Bay (California, U.S)	[80]

**Table 4:** Fatty acids and their derivatives found in cyanobacteria.

Compound	Molecular Weight	Cyanobacterial Strain (Family)	Source	Location	Ref
Mirabilene A isonitrile	407.59	*Scytonema mirabile* BY-8-1 (Scytonemataceae)	Terrestrial	Tantalus (Hawaii, U.S)	[86]
Mirabilene B isonitrile	405.57				
Mirabilene C isonitrile					
Mirabilene D isonitrile					
Mirabilene E isonitrile					
Mirabilene F isonitrile	465.67				
Tanikolide	284.44	*L. majuscula* MNT-6 (Oscillatoriaceae)	Marine	Tanikeli Island (Madagascar)	[88]
Majusculoic Acid	315.25	Environmental Sample	Marine	Sweetings Cay (Bahamas)	[16,85]
		*Aphanothece bullosa* (Microcystaceae)	Freshwater	Banaras Hindu University (India)	
Chlorosphaerolactylate A	341.27	*Sphaerospermopsis* sp. LEGE 00249 (Aphanizomenonaceae)	Freshwater	Maranhão Dam Reservoir (Montargil, Portugal)	[84]
Chlorosphaerolactylate B–C	306.83		Freshwater		
Chlorosphaerolactylate D	375.72		Freshwater		

**Table 5:** List of antifungal alkaloids found in cyanobacteria.

Compound	Molecular Weight	Cyanobacterial Strain (Family)	Source	Location	Ref
Hapalindole A	338.87	*Hapalosiphon fontinalis* ATCC 39694 (Hapalosiphonaceae)	Terrestrial	Marshall Islands	[95]
Hapalindole B	370.94				
Hapalindole C	304.43				
Hapalindole D	336.49				
Hapalindole E	338.87				
Hapalindole F	370.94				
Hapalindole G	338.87				
Hapalindole H	304.43				
Hapalindole X	304.43	*Westiellopsis* sp. SAG 20.93 (Hapalosiphonaceae)	Terrestrial	Mae Hong Son (Thailand)	[93]
Hapalonamide H	336.43				
Hapalindole J	304.43				
Anhydrohapaloxindole A	352.86	*Fischerella muscicola* UTEX LB1829 (Hapalosiphonaceae)	Terrestrial	-	[93]
Fischerindole L	338.87				
12-epi-fischerindole U	304.43	Chemical Synthesis	-	-	[94]
12-epi-fischerindole G	338.87				
13R-Bromo 12-epi-fischerindole U	-				
Fischambiguine A	386.53	*F. ambigua* UTEX 1903 (Hapalosiphonaceae)	Terrestrial	-	[91]
Ambiguine A isonitrile	406.99	*F. ambigua* UTEX 1903 *Hapalosiphon hibernicus* BZ-3-1 *Westiellopsis prolifica* EN-3-1 (Hapalosiphonaceae)	Terrestrial	-	[89,216]
Ambiguine B isonitrile	422.99				
Ambiguine C isonitrile	388.55			Maui Island (Hawaii, U.S)	
Ambiguine D isonitrile	452.97				
Ambiguine E isonitrile	436.98			-	
Ambiguine F isonitrile	454.99				
Ambiguine G isonitrile	402.97	*H. delicatulus* IC-13-1 (Hapalosiphonaceae)	Terrestrial	Australia	[213]
Ambiguine H isonitrile	372.55	*Fischerella* sp. TAU IL-199-3-1 (Hapalosiphonaceae)	Terrestrial	The Cactus Nursery (Herzliya, Israel)	[90]
Ambiguine I isonitrile	402.54				
Ambiguine K isonitrile	420.98	*F. ambigua* UTEX 1903 (Hapalosiphonaceae)	Terrestrial	-	[91,92]
Ambiguine L isonitrile	386.54				
Ambiguine M isonitrile	438.99				
Ambiguine N isonitrile	404.55				
Ambiguine O isonitrile	452.98				
Ambiguine P isonitrile	359.51				
Welwitindolinone A isonitrile	588.98	*Hapalosiphon welwitschii* UH IC-52-3 (Hapalosiphonaceae)	Terrestrial	Australia	[99]
Tjipanazole A	471.33	*Tolypothrix tjipanasensis* DB-1-1 (Tolypothrichaceae)	Terrestrial	Vero Beach (Florida, U.S)	[98]
Carriebowlinol	197.66	*L. majuscula*-*Hormoscilla* sp. Consortium	Marine	Coral reefs (Carrie Bow Cay, Belize)	[87]
Norharmane	168.2	*N. harveyana* 44.85 (Nodulariaceae)	Marine	United Kingdom	[97,217]
		*Synechocystis aquatilis* (Microcystaceae)	Freshwater	Saudi Arabia	[218]
		*Anabaena cylindrica* *SAG 1403-2* (Aphanizomenonaceae)	Freshwater	United Kingdom	[97,217]
		*Anabaena inaequalis* *SAG 1403-10* (Aphanizomenonaceae)	Freshwater	Netherlands	
		*Cylindrospermum* *Siamensis* B 11.82 (Aphanizomenonaceae)	Terrestrial	Thailand	
		*Chroococcus minutus* *SAG 41.79* (Chroococcaceae)	Freshwater	Romania	
		*Nostoc carneum* (Nostocaceae)	-	-	
		*Phormidium foveolarum* UTEX 427 (Oscillatoriaceae)	-	-	
		*Nostoc commune* SAG 1453-5 (Nostocaceae)	-	-	

**Table 6:** List of polyketides identified in cyanobacteria.

Compound	Molecular Weight	Cyanobacterial Strain (Family)	Source	Location	Ref
Kalkipyrone A	332.44	cf. *Leptolyngbya* sp. (Leptolyngbyaceae)	Marine	Fagasa Bay (American Samoa)	[101,235]
		*L. majuscula* and *Tolypothrix* sp. assemblage		Curaçao	
Kalkipyrone B	334.45	cf. *Leptolyngbya* sp. (Leptolyngbyaceae)	Marine	Fagasa Bay (American Samoa)	
Yoshinone A	364.48	cf. *Schizothrix* sp. (Schizotrichaceae)	Marine	Panama	[101]
		*Leptolyngbya* sp. (Leptolyngbyaceae)		Okinawa (Japan)	[236]

**Table 7:** List of Carbamidocyclophanes that exhibit antifungal activity and are isolated from cyanobacteria.

Compound	Molecular Weight	Cyanobacterial Strain	Source	Location	Ref
Carbamidocyclophane A	808.66	*Nostoc* sp. CAVN10	Terrestrial	Vietnam	[240]
		*Nostoc* sp. UIC 10274	Freshwater	Des Plaines (Illinois, U.S)	[102]
		*Nostoc* sp. CAVN02	Freshwater	Vietnam	[239,242]
		*Cylindrospermum* *stagnale* BEA 0605B	Freshwater	Canary Islands (Spain)	[241]
Carbamidocyclophane B	774.21	*Nostoc* sp. CAVN10	Terrestrial	Vietnam	[240]
		*Nostoc* sp. UIC 10274	Freshwater	Des Plaines (Illinois, U.S)	[102]
		*Nostoc* sp. CAVN2	Freshwater	Vietnam	[242]
Carbamidocyclophane F	765.63	*Nostoc* sp. UIC 10274	Freshwater	Des Plaines (Illinois, U.S)	[102]
		*Nostoc* sp. CAVN2	Freshwater	Vietnam	[242]
		*Cylindrospermum* *stagnale* BEA 0605B	Freshwater	Canary Islands (Spain)	[241]

**Table 8:** List of macrolides obtained from cyanobacteria.

Compound	Molecular Weight	Cyanobacterial Strain (Family)	Source	Location	Ref
Scytophycin A	822.08	*S*. *pseudohofmanni* ATCC 53141 (Scytonemataceae)	Terrestrial	Island of Oahu (Hawaii, U.S)	[103]
Scytophycin B	820.07	*Cylindrospermum muscicola* GO-17-1	Terrestrial	Island of Kauai (Hawaii, U.S)	
		*S*. *pseudohofmanni* ATCC 53141	Terrestrial	Island of Oahu (Hawaii, U.S)	
Scytophycin C	806.09	*S. pseudohofmanni* ATCC 53141	Terrestrial	Island of Oahu (Hawaii, U.S)	[103]
		*Scytonema* sp. UIC 10036	Freshwater	Homestead (Florida, U.S)	[252]
Scytophycin D	822.09	*S. pseudohofmanni* ATCC 53141	Terrestrial	Island of Oahu (Hawaii, U.S)	[103]
Scytophycin E	822.09	*C. muscicola* GO-17-1	Terrestrial	Island of Kauai (Hawaii, U.S)	[103]
		*S. pseudohofmanni* ATCC 53141	Terrestrial	Island of Oahu (Hawaii, U.S)	[103]
Tolytoxin	850.09	*Scytonema ocellatum* FF-66-3	-	South Pasture Pond (Illinois, U.S)	[104]
		*S. ocellatum* FF-65-1	-	Columbia (Missouri, U.S)	
		*S. ocellatum* DD-8-1	-	University of Guam (Guam)	
		*S. mirabile* BY-8-1	Terrestrial	Island of Oahu (Hawaii, U.S)	
		*S. burmanicum* DO-4-1	-	Moon Beach (Okinawa, Japan)	
		*Scytonema* sp. UIC 10036	Freshwater	Homestead (Florida, U.S)	[252]
Swinholide A	1389.89	*Symploca* cf. sp. (Phormidiaceae)	Marine	Fiji Islands	[248]
		*Geitlerinema* sp. (Coleofasciculaceae)		Nosy Mitsio (Madagascar)	
Amantelide A	789.14	A Member of *Oscillatoriales* family	Marine	Puntan dos Amates (Guam)	[106]
Sacrolide A	308.41	*A. sacrum* (Microcystaceae)	Freshwater	Kyushu District (Japan)	[107,108]
9-epi-sacrolide A	308.41				
15,16-dihydrosacrolide A	310.43				

**Table 9:** Phenolic compounds and other metabolites exhibiting antifungal activity from cyanobacteria.

Compound	Molecular Weight	Cyanobacterial Strain (Family)	Source	Location	Ref
4,4′-dihydroxybiphenyl	186.21	*Nostoc insulare* SAG 54.79 (Nostocaceae)	Terrestrial	-	[97]
Ambigol A	484.97	*Symphyonema bifilamentata* sp. nov. 97.28 (Symphyonemataceae)	Terrestrial	Mellingen (Switzerland)	[109,258]
Ambigol C	484.97				
Scytoscalarol	415.66	*Scytonema* sp. UTEX 1163 (Scytonemataceae)	Terrestrial	-	[111]
Parsiguine	160.00	*F. ambigua* (Hapalosiphonaceae)	Terrestrial	Noushahr (Iran)	[110]

## Data Availability

Not applicable.
